# Real-time tracking of bioluminescent influenza A virus infection in mice

**DOI:** 10.1038/s41598-022-06667-w

**Published:** 2022-02-24

**Authors:** Jin H. Kim, Hannah Bryant, Edward Fiedler, TuAnh Cao, Jonathan O. Rayner

**Affiliations:** 1 Department of Microbiology and Immunology, College of Medicine, University of South Alabama, Mobile, AL 36688 USA; 2 Center for Lung Biology, College of Medicine, University of South Alabama, Mobile, AL 36688 USA; 3 Department of Comparative Medicine, College of Medicine, University of South Alabama, Mobile, AL 36688 USA

**Keywords:** Virology, Influenza virus

## Abstract

Despite the availability of vaccines and antiviral therapies, seasonal influenza infections cause 400,000 human deaths on average per year. Low vaccine coverage and the occurrence of drug-resistant viral strains highlight the need for new and improved countermeasures. While influenza A virus (IAV) engineered to express a reporter gene may serve as a valuable tool for real-time tracking of viral infection, reporter gene insertion into IAV typically attenuates viral pathogenicity, hindering its application to research. Here, we demonstrate that lethal or even sublethal doses of bioluminescent IAV carrying the NanoLuc gene in the C-terminus of PB2 can be tracked in real-time in live mice without compromising pathogenicity. Real-time tracking of this bioluminescent IAV enables spatiotemporal viral replication tracking in animals that will facilitate the development of countermeasures by enhancing the interpretation of clinical signs and prognosis while also allowing less animal usage.

## Introduction

The number of seasonal influenza-related deaths worldwide is estimated between 290,000 and 645,000 per year^1^. Seasonal vaccination is the primary method to control and prevent influenza. Although influenza virus vaccines show excellent efficacy in preclinical studies, the effectiveness of seasonal influenza vaccines remains at the range of 19–60% over a 10-year period^2^, highlighting the need for improving the vaccine. Together with the continuous threat of emerging flu pandemics, therapeutic antiviral drugs including neuraminidase (NA) inhibitors, M2 ion channel blockers, viral RNA polymerase inhibitors, and viral endonuclease inhibitors have been developed as countermeasures. These antivirals, however, still need to overcome limitations such as a narrow administration window for effectiveness and proclivity for the emergence of drug-resistant viruses, thus highlighting the need for new or improved countermeasures to combat this continued threat to human life.

In vivo imaging of live animals enables real-time monitoring of viral spread and may facilitate new vaccine and antiviral drug development against influenza A virus (IAV). Viral tracking with in vivo imaging may enable dynamic and longitudinal studies about prophylaxis and therapy that are not feasible in a terminal animal model. Specifically, tracking of IAV in animals empowers us to investigate various factors critical to viral replication such as host factors, transmissibility, pathogenicity, and coinfections in a real-time fashion. Importantly, it can also be utilized to reduce the number of animals used in basic and preclinical research by minimizing the need for serial sacrifice studies.

Several attempts to establish IAVs carrying fluorescent or bioluminescent proteins for in vivo studies have been made by inserting a reporter gene into nonstructural protein genes such as polymerase basic protein 2 (PB2), polymerase acidic protein (PA), or nucleoprotein (NP)^3–7^; or structural protein genes such as hemagglutinin (HA) or neuraminidase (NA)^8^. However, these reporter IAV constructs suffered from diminished replicative ability resulting in attenuation in animal models^3–5,7–9^. Although pathogenicity could be recovered by serial passage in mice^7,10^, the inclusion of adaptive mutations makes it difficult to compare results with published data, thereby limiting the utility of these reporter IAVs in replication and transmission studies. In addition, it is crucial to understand how sensitively the replication of reporter viruses can be tracked during in vivo imaging; however, such characterization is limited with the previously developed IAV reporter viruses.

NanoLuc is a luciferase reporter that is derived from deep-sea shrimp (*Oplophorus gracilirostris*), has 150 times brighter luminescence than the commonly used Renilla and firefly luciferase reporters, and has been used previously in reporter IAVs for in vivo imaging^4–6,9–11^. Gaussia luciferase (GLuc) is another bright luciferase used for in vivo imaging of reporter IAVs^3,8,12^ but has intrinsic autoluminescence that decreases bioluminescence detection in animals making it less sensitive for generating reporter viruses^13^. As an alternative, NanoLuc Binary Technology (NanoBiT) has been developed as a complementation reporter which includes a small subunit tag (11 amino acids) known as *high-affinity NanoBiT* (HiBiT) and a larger subunit known as *large NanoBiT* (LgBiT)^14^. When LgBiT binds to HiBiT, the combined protein functions like NanoLuc in bioluminescent characteristics. Inserting the small HiBiT tag into the viral genome is less likely to interfere with viral replication but will produce strong bioluminescence when LgBiT is provided exogenously as has been demonstrated previously with viruses in the *Flaviviridae* family^15^. A search of the literature identified no previous studies using HiBiT with IAV.

We hypothesized that the NanoLuc or HiBiT reporters may be inserted into the IAV genome without loss of virulence thus enabling in vivo bioluminescence towards the study of IAV infection in live animals. The present study aimed to evaluate the bioluminescent reporter gene (NanoLuc) and tag (HiBiT) inserted into the IAV genome. To generate bioluminescent IAV, we inserted the reporters in the background of influenza A/Puerto Rico/8/1934, H1N1 virus (PR8). Here, we showed that insertion of NanoLuc into the C terminus of the PB2 gene resulted in a virus (PB2-C-NanoLuc PR8 IAV) that had comparable virulence with wild-type IAV in mice and sufficient detection sensitivity in the respiratory tract to enable in vivo viral tracking, even at sublethal doses. This suggests that the PB2-C-NanoLuc PR8 IAV will be a useful real-time tracking reporter virus for basic and applied IAV research.

## Results

### Comparison of bioluminescent IAVs carrying the NanoLuc gene or HiBiT tag

**Figure 1:**
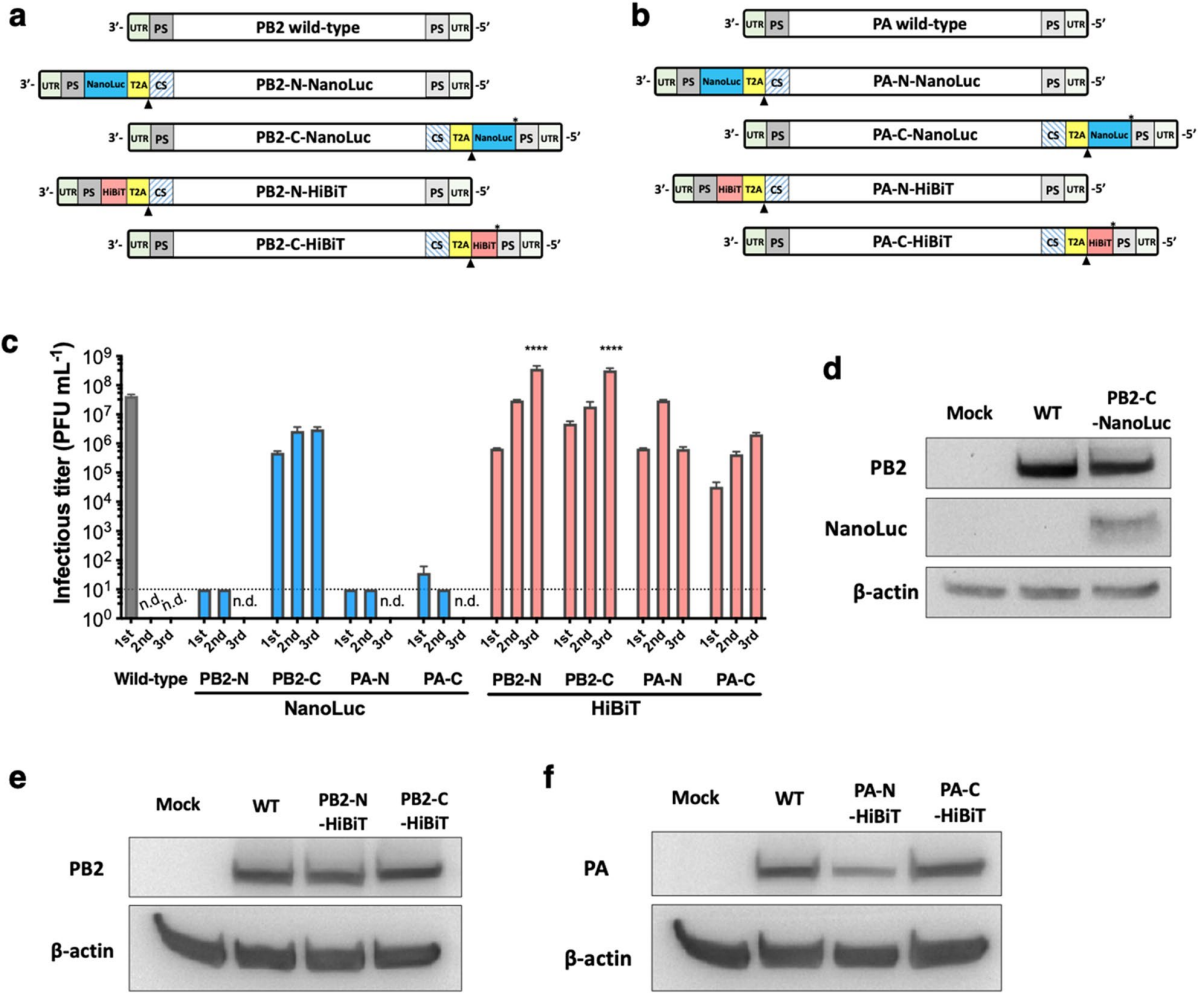
Generation of bioluminescent PR8 IAV carrying NanoLuc or HiBiT reporters. (**a,b**) Schematic of viral RNA (vRNA) segments in bioluminescent IAV. NanoLuc (510 nt, blue) or HiBiT (33 nt, pink) was inserted into the N- or C-terminus of IAV protein-coding regions linked by the T2A self-cleaving peptide sequence (63 nt, yellow). Native packaging signal (PS) sequences adjacent to the T2A sequence were replaced with codon swapped (CS) sequences. Diagrams are not to scale. Modified sequences in PB2 and PA segments are presented in Supplementary Fig. 8a,b. (**a**) PB2 vRNA segments. 3′-PB2 fragments contained 3′-UTR (27 nt), 3′-minimum PS sequence (120 nt), NanoLuc or HiBiT sequence, T2A sequence, and 3′ CS sequence (67% of nt homology to PB2). 5′-PB2 fragments included 5′ CS sequence (66% of nt homology to PB2) where the stop codon was removed, T2A sequence, T2A cleavage site (black triangle), NanoLuc or HiBiT sequence, stop codon (*), 5′-minimum PS sequence (120 nt), and 5′-UTR (34 nt). (**b**) PA vRNA segments. 3′-PA fragments contained 3′-UTR (24 bp), 3′-minimum PS sequence (60 nt), NanoLuc or HiBiT sequence, T2A sequence, and 3′ CS sequence (71% of nt homology to PA). 5′-PA fragments included 5′ CS sequence (75% of nt homology to PA) where the stop codon was removed, T2A sequence, NanoLuc or HiBiT sequence, stop codon (*), 5′-minimum PS sequence (60 nt), and 5′-UTR (58 nt). (**c**) Rescue of bioluminescent IAVs and serial passages in MDCK cells at low multiplicity of infection (MOI, 0.001) and 37 °C. Data reported as mean ± SD of results in triplicate for first, second, and third passages. Viral infectious titers shown for wild-type (WT) IAV (gray) or IAVs carrying NanoLuc (blue) or HiBiT (pink). *n.d.* not determined. Dotted line indicates limit of detection (10 PFU mL^−1^). ****p < 0.0001. (**d–f**) Protein expression from bioluminescent IAV-infected MDCK cells. Whole MDCK cell lysate with mock infection or infection with WT or bioluminescent IAV (MOI, 0.001), collected at 24 h post inoculation, and examined by Western blot using specific antibodies for PB2, PA, NanoLuc, and β-actin. Cropped blots are displayed. Full-length blots are presented in Supplementary Fig. 9a,b. (**d**) PB2-C-NanoLuc. (**e**) PB2-N-HiBiT and PB2-C-HiBiT. (**f**) PA-N-HiBiT and PA-C-HiBiT.

To investigate an IAV with a reporter that retained viral replication like wild-type PR8 IAV, the NanoLuc gene or HiBiT tag was inserted into the N- and C-terminal ends of the PB2 or PA gene segment coding regions (Fig. 1a). These gene segments have previously been demonstrated to tolerate reporter genes in the C-terminus^3,4,6,16,17^, though the resulting IAVs had decreased virulence^3,4,16^. For the PB2 gene segment, 120 nucleotides (nt) at the N- and C-terminal coding regions are required to maintain packaging efficiency, while 60 nt are required for the PA gene segment^3,6,18,19^ (Fig. 1a,b). To ensure stability of the NanoLuc gene or HiBiT tag, the original packaging signal sequence within the coding region was swapped, but the amino acid sequence was maintained by introducing silent mutations. For the bicistronic expression of NanoLuc or HiBiT with PB2 or PA, the *Thosea asigna* virus self-cleaving 2A peptide sequence (T2A) (length, 21 amino acids) was used because of its efficient expression, cleavage, and localization of the second gene in contrast with the commonly used P2A peptide sequence from Porcine teschovirus-1^20–22^. When T2A self-cleavage occurs via “ribosomal-skip or STOP&GO mechanism during translation”^21^, the first protein contains a fusion of 20 amino acids. Therefore, additional N-terminal tagged versions of PB2 or PA gene segments were constructed to discern the effect of residual amino acids on growth properties.

We attempted to rescue 4 bioluminescent PR8 IAVs containing NanoLuc and 4 HiBiT-tagged IAVs with insertions in the PB2 or PA gene segments as described above using reverse genetics^23^ and propagate them in Madin-Darby Canine Kidney (MDCK) cells. PB2-C-NanoLuc and all 4 HiBiT-tagged PR8 IAVs (PB2-N-HiBiT, PB2-C-HiBiT, PA-N-HiBiT, and PA-C-HiBiT) were rescued successfully and maintained infectivity in MDCK cells after 3 passages (Fig. 1c). Attempts to rescue N-terminal NanoLuc variants from PB2 and PA segments were unsuccessful after 3 attempts. The PA-C-NanoLuc IAV grew just above the limit of detection in MDCK cells but lost infectivity during the second passage (Fig. 1c). These results suggest that the longer length of the NanoLuc gene (510 nt) as compared to the HiBiT tag negatively affected PB2 or PA gene segment packaging. In support of this assumption, IAVs tagged at the PB2-C terminus with longer reporters such as monomeric oxidizing environment-optimized green fluorescent protein (moxGFP; 714 nt)^23^ and improved Causes recombination (iCre; 1050 nt)^24^, had lower infectious titers than PB2-C-NanoLuc IAV (Supplementary Fig. 1).

To verify incorporation of NanoLuc or HiBiT-tagged segment (PB2 or PA) into the virus, MDCK cells were infected with rescued IAVs (PB2-C-NanoLuc and all 4 HiBiT-tagged viruses) at low multiplicity of infection (MOI, 0.001), and cell lysates were collected at 1 day after inoculation. MDCK cells infected with PB2-C-NanoLuc IAV expressed PB2 and NanoLuc proteins confirming that the NanoLuc gene was incorporated into virions (Fig. 1d). Similarly, PB2-N-HiBiT, PB2-C-HiBiT, PA-N-HiBiT, and PA-C-HiBiT segments were expressed in MDCK cells infected with these reporter IAVs (Fig. 1e,f). Incorporation of HiBiT tagged segments was confirmed by luciferase assay in MDCK cells after infection (Fig. 3, Supplementary Fig. 2).

### Growth properties of PB2-C-NanoLuc and HiBiT-tagged IAVs

**Figure 2:**
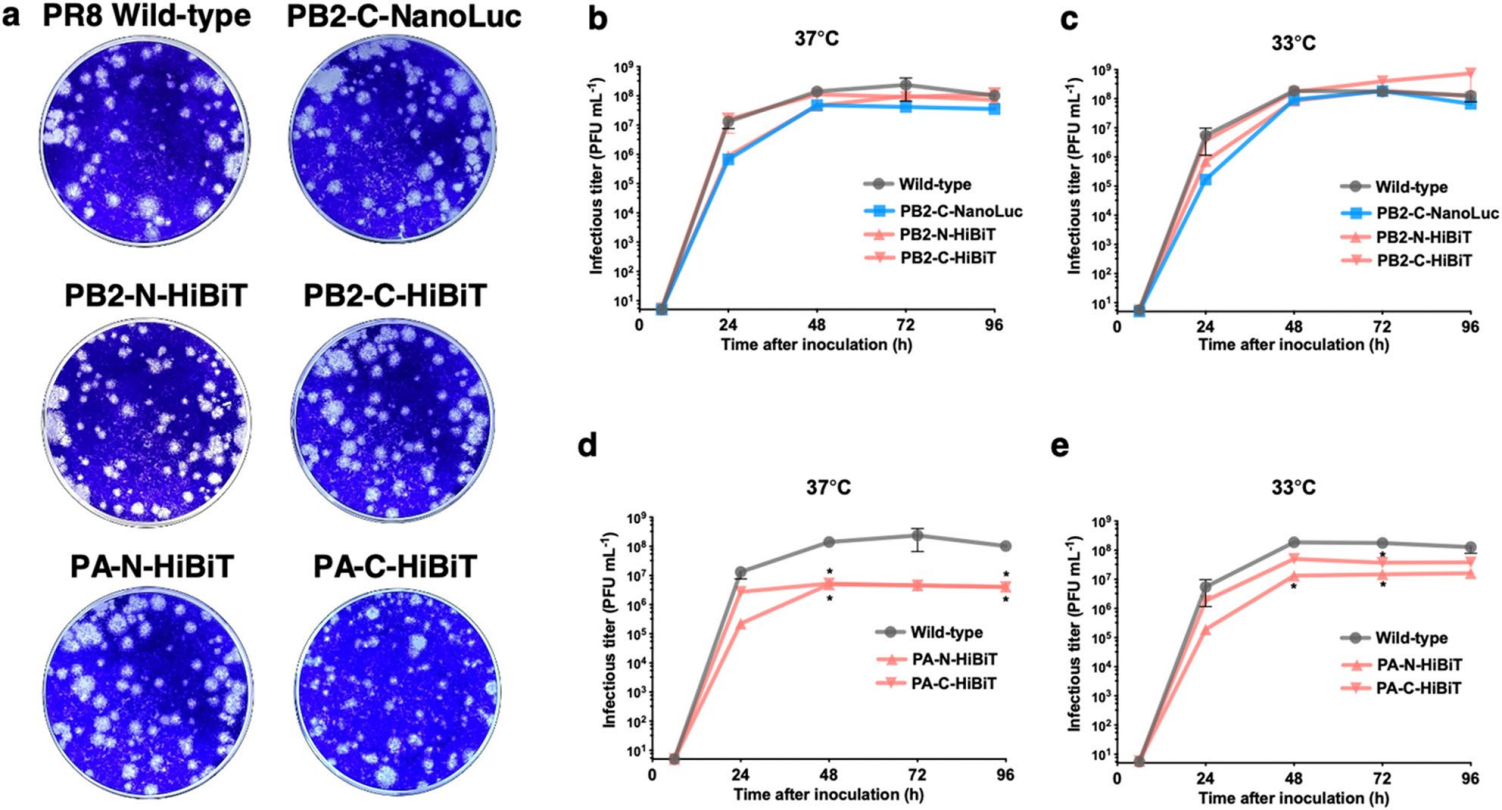
Growth kinetics of bioluminescent IAVs in MDCK cells. (**a**) Plaque formation of bioluminescent IAVs in MDCK cells. We compared plaque formation from bioluminescent IAVs at 4 days post inoculation. Confluent MDCK cells in 12-well plates were infected with IAVs (MOI, 0.0002), and plaques were stained with crystal violet, and imaged. PB2-C-NanoLuc and all HiBiT IAVs formed plaques like wild-type PR8 IAV, indicating that the NanoLuc or HiBiT tags did not hinder plaque formation of bioluminescent IAVs. (**b,c**) Multi-step growth curves of PB2-C-NanoLuc (blue), PB2-N-HiBiT and PB2-C-HiBiT (pink), and wild-type PR8 IAVs (gray) at low multiplicity of infection (MOI, 0.001). (**b**) At 37 °C, all PB2 bioluminescent IAVs replicated productively, like wild-type PR8 IAV. (**c**) At 33 °C, all PB2 bioluminescent IAVs replicated productively, like wild-type PR8 IAV. (**d,e**) Multi-step growth kinetics of PA-N-HiBiT and PA-C-HiBiT (pink) and wild-type PR8 IAVs (gray) at low MOI (0.001). (**d**) At 37 °C, PA-N-HiBiT and PA-C-HiBiT replication were significantly less than wild-type IAV. *p < 0.05. (**e**) At 33 °C, PA-N-HiBiT and PA-C-HiBiT replication were significantly less than wild-type IAV. Data represent the mean ± SEM of results determined in 3 independent experiments, each performed in triplicate. The limit of detection was 10 PFU mL^−1^. *p < 0.05.

To access the replicative ability of rescued bioluminescent IAVs, plaque formation of reporter IAVs was compared with wild-type PR8 IAV in MDCK cells. PB2-C-NanoLuc and all HiBiT IAVs visually formed plaques like wild-type PR8 IAV, indicating that NanoLuc or HiBiT tag did not hinder the ability of bioluminescent IAVs to form plaques (Fig. 2a). To ascertain that the viral plaques contain NanoLuc protein, the viral plaques of PB2-C-NanoLuc IAV were immunostained with anti-NP and anti-NanoLuc antibodies. Among 105 PB2-C-NanoLuc IAV plaques/foci immunostained with anti-NP antibody, 103 plaques/foci were immunostained with anti-NanoLuc antibody. The two plaques/foci which express only NP but not NanoLuc were tiny foci (3–4 cells stained with NP), thus it is likely that they originated from defective particles which didn’t contain PB2-C-NanoLuc segments. This observation is consistent with a previous study demonstrating that about 90% of IAV particles contained both NP and PB2 segments^25^. To further investigate growth properties of rescued bioluminescent IAVs, multi-step replication of reporter IAVs was compared with wild-type PR8 IAV in MDCK cells at low MOI (0.001) and 37 °C vs. 33 °C to simulate replication in the mouse upper respiratory tract^26^. All of the PB2 bioluminescent IAVs replicated similarly to wild-type PR8 IAV, while PA-N-HiBiT and PA-C-HiBiT replication was significantly decreased as compared to wild-type (Fig. 2b–e). The T2A self-cleaving sequence in the bicistronic expression system cleaves into a 20 amino acid segment that fuses to the first bicistronic protein while 1 amino acid residue fuses to the second bicistronic protein^22^. Throughout the multi-step replication experiments, no significant difference in replication was observed between N-terminal and C-terminal PB2 HiBiT tagged IAVs (Fig. 2b,c), suggesting that the 20 amino acid residues in the C terminus of the PB2 protein following T2A self-cleavage did not hamper viral replication. Since replication of PB2-C-NanoLuc, PB2-N-HiBiT, and PB2-C-HiBiT was most like wild-type PR8 IAV at 37 °C and 33 °C, we concluded that IAVs tagged in the PB2 gene segment, but not the PA gene segment, were most likely to exhibit near wild-type virulence in mice and only these 3 bioluminescent IAVs were assessed in subsequent studies.

### Stability of NanoLuc and HiBiT

**Figure 3:**
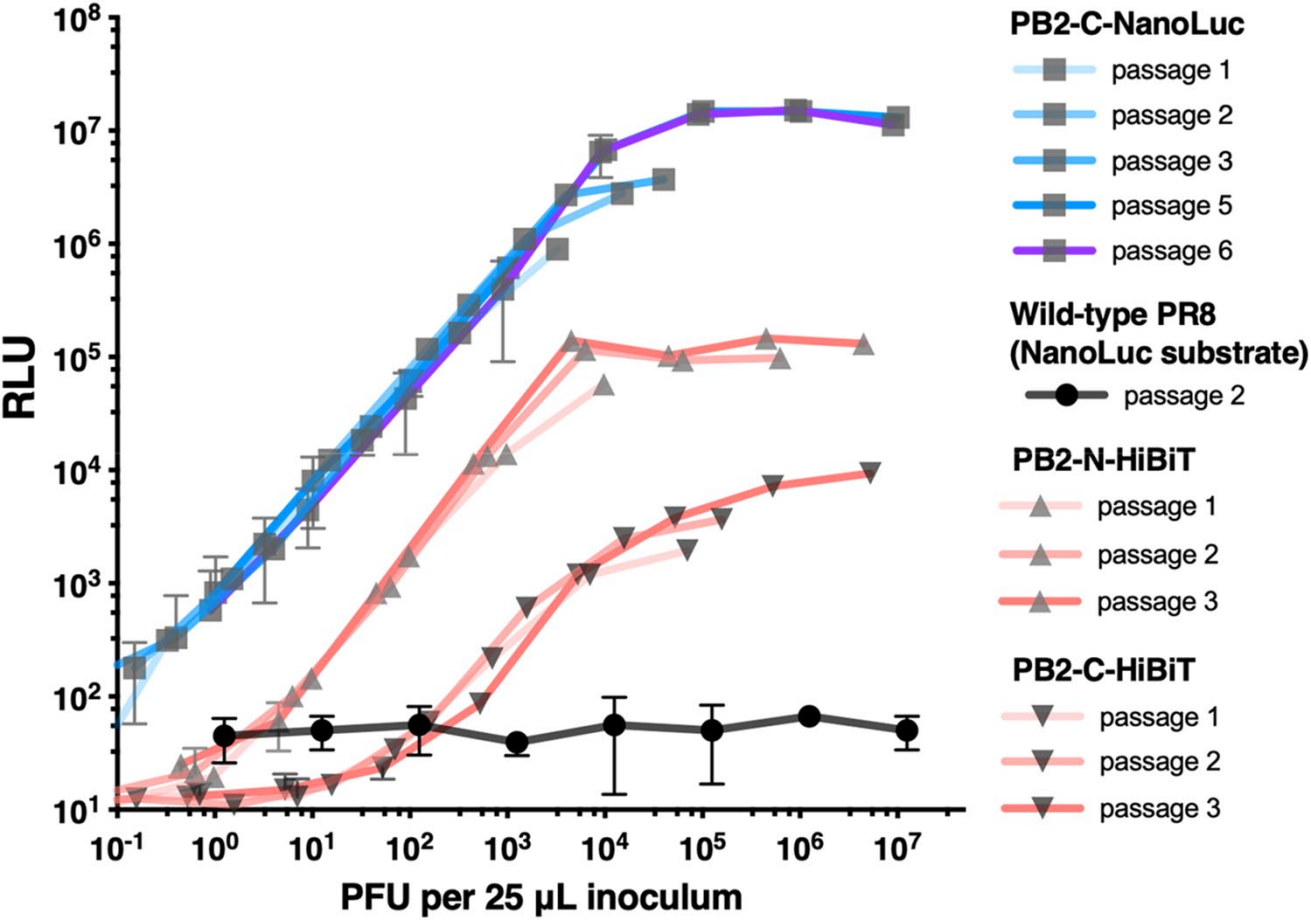
Functional stability of luciferase activity in bioluminescent IAVs after sequential passage. PB2-C-NanoLuc (blue) and PB2-N-HiBiT and PB2-C-HiBiT (pink) IAVs were sequentially passaged in MDCK cells at 37 °C. Each passaged IAV was serially diluted, and IAV luciferase activity was determined in MDCK cells at 12 h after inoculation. Stability of NanoLuc or HiBiT tag in PB2-C-NanoLuc, PB2-N-HiBiT, and PB2-C-HiBit was confirmed after 3–6 sequential passages of these IAVs in MDCK cells. RLU with NanoLuc substrate from MDCK cells infected with wild-type PR8 IAV (black) was plotted as a reference. Data reported as mean ± SD (N = 3). *PFU* plaque forming units, *RLU* relative luciferase units.

Although transgenes flanked by repeated packaging signal sequences in IAVs may be unstable and lost during replication^27,28^, the instability could be resolved by introducing silent mutations in the coding region of the packaging signal sequence^3,6^. Therefore, the packaging signal sequence in the coding region of bioluminescent IAVs was codon swapped (Fig. 1a,b). Sequencing of serially passaged PB2-C-NanoLuc IAV showed that the NanoLuc gene was stably maintained until the sixth passage, when a single amino acid change was introduced at P115S (Table 1). For PB2-N-HiBiT and PB2-C-HiBiT, the PB2 gene sequence was stably maintained through 5 passages and HiBiT through 6 passages in MDCK cells. As point mutations in the HA gene were observed in the third passage in MDCK cells, all stocks of reporter viruses were prepared before the third passage (Table 1). Stability of NanoLuc or HiBiT tag in PB2-C-NanoLuc, PB2-N-HiBiT, and PB2-C-HiBit was further confirmed after 3 sequential passages in MDCK cells by demonstrating that functional luciferase activity was preserved (Fig. 3).

**Table 1:** Mutations in bioluminescent influenza A viruses sequentially passaged in MDCK cells.

Bioluminescent IAV	Passage no. in MDCK cells	PB2	NanoLuc or HiBiT	PB1	PA	HA	NP	NA	M	NS
**PB2-C-NanoLuc**	3	–	–	–	–	–	–	–	–	–
	5	D710N	–	–	n.d.	–	n.d.	n.d.	–	–
	6	D710N	P115S	–	–	I353L	–	–	–	–
**PB2-N-HiBiT**	3	–	–	–	–	–	–	–	–	–
	5	–	–	–	n.d.	T450I	n.d.	n.d.	–	–
**PB2-C-HiBiT**	3	–	–	–	–	D455N	–	–	–	–
	5	–	–	–	n.d.	D455N	n.d.	n.d.	–	–
	6	–	–	–	–	D455N	–	–	–	–

**− ** No mutation identified, ***n.d.*** not determined.

### In vivo imaging of PB2-C-NanoLuc, PB2-N-HiBiT, and PB2-C-HiBiT IAVs

**Figure 4:**
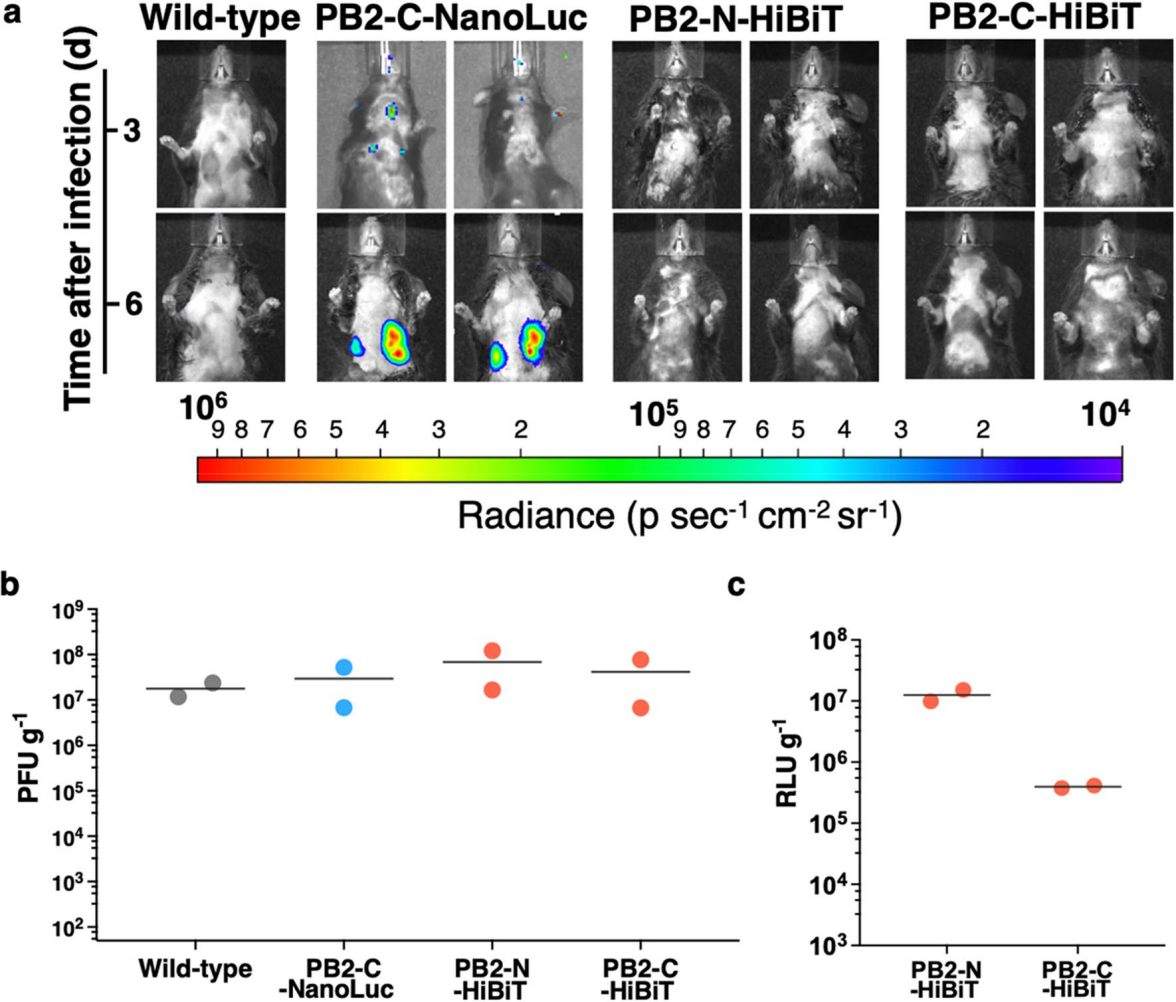
In vivo imaging of PB2-C-NanoLuc, PB2-N-HiBiT, and PB2-C-HiBiT IAVs. Female C57BL/6 mice aged 6 to 8 weeks were infected with 2000 PFU of wild-type PR8 or 20,000 PFU of PB2-C-NanoLuc, PB2-N-HiBiT, or PB2-C-HiBiT IAVs intranasally (2 mice per each virus). Luciferase substrates for PB2-C-NanoLuc or PB2-N-HiBiT and PB2-C-HiBiT were prepared and injected retro-orbitally for imaging. The mice were imaged using an in vivo imaging system (IVIS) and viral titers were determined in lung homogenates prepared from euthanized animals on day 6 post challenge. Black lines, mean values. (**a**) IVIS imaging from NanoLuc or HiBiT-LgBiT complex at 3 and 6 days post infection. Radiance, defined as the number of photons per s per square cm per steradian (p s^−1^ cm^−2^ sr^−1^), is shown on the heat maps. (**b**) In vitro viral titers in lung homogenates determined by plaque assay (PFU g^−1^). The limit of detection: nasal turbinates, 136 PFU g^−1^; trachea, 188 PFU g^−1^; lung, 65 PFU g^−1^. (**c**) In vitro viral titers in lung homogenates from PB2-N-HiBiT, or PB2-C-HiBiT IAV infected mice determined by luciferase assay on MDCK cells. The infected lung homogenates showed active luciferase signals, indicating that HiBiT peptide carried by IAVs caused luciferase activity when substrate and LgBiT protein were supplemented. The limit of detection in lung, 34,783 RLU (MDCK) g^−1^. *PFU* plaque forming units, *RLU* relative luciferase units.

To determine which of the 3 remaining bioluminescent PR8 IAVs were most efficiently tracked in vivo, real-time imaging of bioluminescent IAVs was performed at 3 and 6 days after intranasal challenge. Bioluminescent signal from PB2-C-NanoLuc was localized to the trachea and both lungs at 3 days post challenge and spread to a wide area of the left lung while disappearing from the trachea at 6 days (Fig. 4a). In contrast, no bioluminescence was detected in mice infected with PB2-N-HiBiT or PB2-C-HiBiT at 3 or 6 days post challenge, even when the amount of LgBiT protein was doubled (Fig. 4a). Control mice infected with wild-type PR8 IAV did not show any bioluminescence as was expected (Fig. 4a).

To confirm that reporter IAVs replicated in mice, infected mice were euthanized after imaging at 6 days post challenge and infectious titers were determined in lung tissue by plaque assay. All 3 bioluminescent IAVs had similar titers in lung tissue as compared to wild-type PR8 IAV (Fig. 4b). Furthermore, HiBiT-tagged IAVs that were collected from lung tissue exhibited luciferase activity upon infection of MDCK cells and exposure to HiBiT substrate and LgBiT protein (Fig. 4c), suggesting that the absence of bioluminescence with PB2-N-HiBiT and PB2-C-HiBiT in vivo was due to insufficient delivery of LgBiT protein to infected cells in the trachea and lungs.

### Virulence of bioluminescent PB2-C-NanoLuc IAV

**Figure 5:**
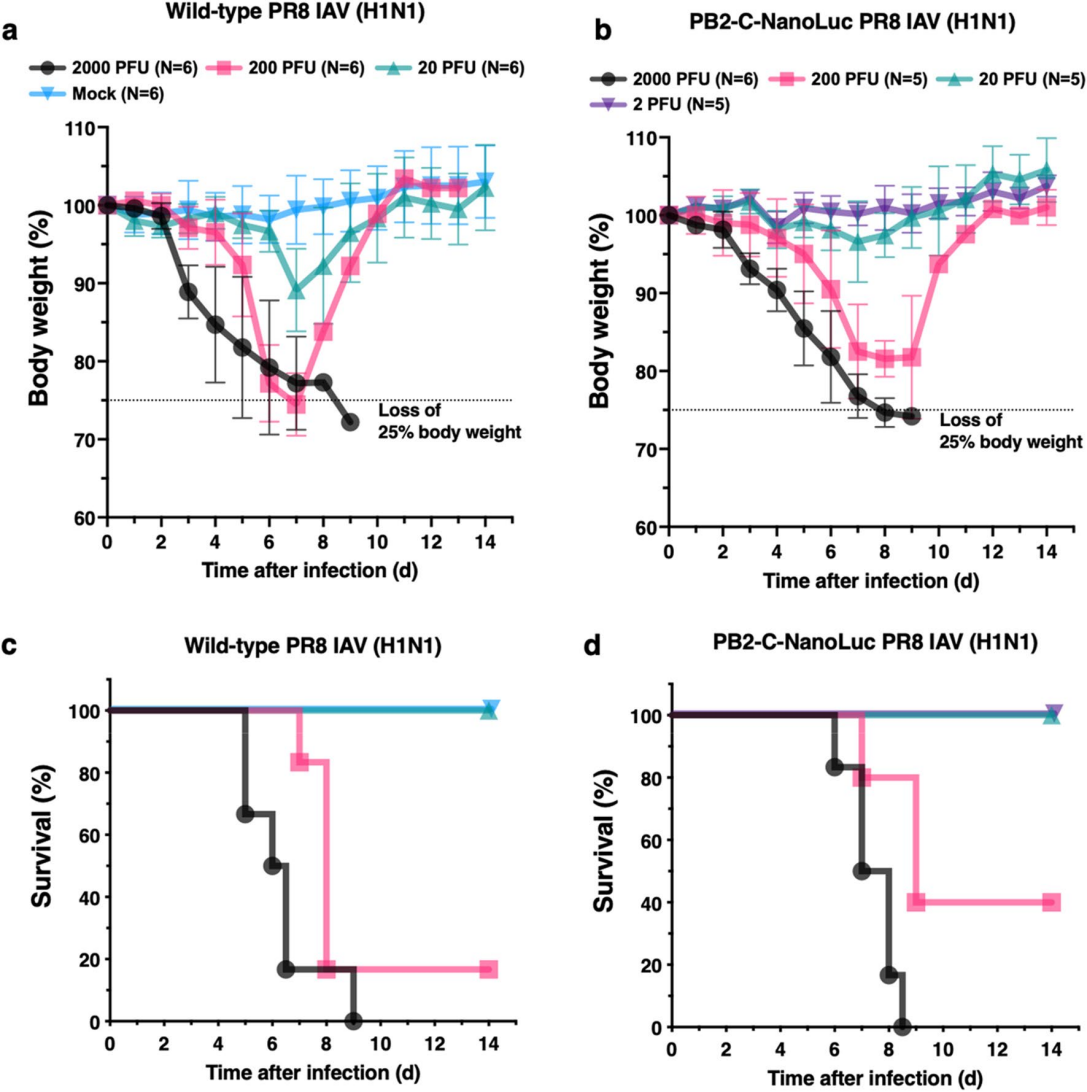
Virulence of wild-type and PB2-C-NanoLuc PR8 IAVs in mice. Female C57BL/6 mice aged 6 to 8 week were infected intranasally with wild-type or PB2-C-NanoLuc PR8 IAV at various doses or no virus (mock) in sterile PBS and observed for 14 days. Body weight reported as percentage of initial body weight (mean ± SD). (**a**) Body weight in cohorts of mice given mock infection or infected with wild-type PR8 IAV. Body weight decreased initially in infected mice but increased after 7 days in mice infected with 20 or 200 PFU of wild-type IAV that survived. (**b**) Body weight change in cohorts of mice infected with PB2-C-NanoLuc PR8 IAV. Body weight decreased initially in infected mice but increased in mice infected with 20 or 200 PFU of PB2-C-NanoLuc IAV that survived beyond 7 to 9 days. (**c**) Survival in mice given mock infection or infected with wild-type PR8 IAV; 5 of 6 mice infected with 200 PFU (83%) died, and all 6 mice infected with 2000 PFU (100%) died within 9 days. The 50% mouse lethal dose (MLD_50_) of wild-type PR8 IAV was 95 PFU. (**d**) Survival in mice infected with PB2-C-NanoLuc PR8 IAV; 3 of 5 mice infected with 200 PFU (60%) died, and all 6 mice infected with 2000 PFU (100%) died within 8.5 days. The MLD_50_ of PB2-C-NanoLuc PR8 IAV was 160 PFU. Log-rank test showed no significant difference between survival curves from wild-type vs PB2-C-NanoLuc IAV at 200 PFU (*p* = 0.26) and 2000 PFU (*p* = 0.42). Time to loss of 25% of initial body weight was similar between mice challenged with wild-type (200 PFU, 7–8 days post infection; 2000 PFU, 5–9 days post infection) and PB2-C-NanoLuc PR8 IAV (200 PFU, 7–9 days post infection; 2000 PFU, 6–8.5 days post infection). *PFU* plaque forming units.

Given that PB2-C-NanoLuc PR8 IAV had optimal imaging results for real-time tracking in vivo, we evaluated maintenance of pathogenicity by challenging mice with tenfold serial dilutions of virus. Although mock-infected mice or mice infected with 2 PFU of PB2-C-NanoLuc IAV gained body weight over the 14 days in life period, mice infected with 20 or 200 PFU of wild-type or PB2-C-NanoLuc IAV lost body weight during the first week after infection (Fig. 5a,b). Most mice infected with PR8 IAV at 200 PFU and all mice infected with 2000 PFU of wild-type or PB2-C-NanoLuc IAV were moribund and euthanized (Fig. 5c,d). Survival curves were similar for mice infected with wild-type or PB2-C-NanoLuc PR8 IAV (Fig. 5c,d) supporting that the insertion of the NanoLuc gene linked with a T2A cleavage sequence in the C terminus of PB2 did not attenuate the virulence of PR8 IAV in mice.

### Real-time tracking of PB2-C-NanoLuc IAV in the mouse respiratory tract

**Figure 6:**
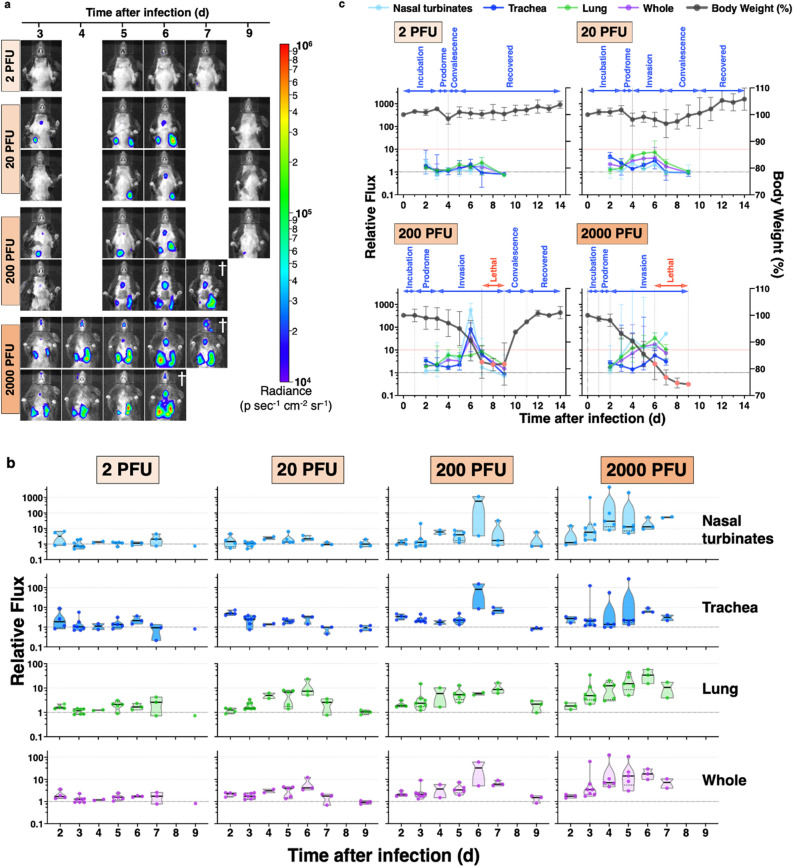
Real-time in vivo tracking of bioluminescent PB2-C-NanoLuc IAVs and spatiotemporal resolution of IAV replication kinetics in the mouse respiratory tract. (**a**) Bioluminescence from mice infected with PB2-C-NanoLuc IAV (2, 20, 200, or 2000 PFU) in the virulence study from 3 to 9 days after infection, expressed as radiance (p s^−1^ cm^−2^ sr^−1^). Each row of images was acquired longitudinally from 1 mouse. Differences in bioluminescence between mice were observed in the nasal turbinates (2000 PFU), trachea (200 PFU), and right or left lung (20 PFU). ^†^Imaging performed before mouse was euthanized because of loss of > 25% of initial body weight. (**b**) Replication kinetics of bioluminescent PB2-C-NanoLuc IAV in mice at different infection doses. Relative bioluminescence flux at each region was the bioluminescence photon flux normalized to the mean flux from 3 mock-infected mice at the nasal turbinates (3.0 × 10^3^ ± 8.7 × 10^2^ photons s^−1^), trachea (2.4 × 10^4^ ± 3.6 × 10^3^ photons s^−1^), lung (5.8 × 10^4^ ± 7.4 × 10^3^ photons s^−1^), and whole body including head and torso (1.2 × 10^5^ ± 1.2 × 10^4^ photons s^−1^). Additional measurements were done at 2 and 5 days after infection to fill gaps resulting from the limited retro-orbital administration of substrate. There were 1 to 7 mice imaged at each time, except that there were no surviving mice at 9 days after infection with 2000 PFU. Data are shown as truncated violin plots with median (line) and first and third quartiles (dashed lines). (**c**) Relative flux for each IAV dose from the nasal turbinates, trachea, lung, and whole-body (median ± 95% confidence interval). Body weight (mean ± SD) after infection with bioluminescent IAV was replotted from Fig. 5b to facilitate comparison of the time course of relative flux and body weight. The natural history of influenza viral infection and progression in animals included incubation (no body weight drop after infection), prodrome (initial body weight drop), invasion (rapid or continuous loss of body weight), and convalescence periods (gaining of body weight) and recovered healthy condition (steady status of body weight). The lethal phase (red points) was defined by the loss of more than 25% of initial body weight.

To monitor how bioluminescent imaging in vivo correlated with body weight as a proxy for the presence of IAV infection, bioluminescence imaging was performed over time in mice infected with PB2-C-NanoLuc IAV. Representative bioluminescence images from infected mice showed variation in spatiotemporal bioluminescence in vivo, suggesting that IAV replication varied with time and anatomical respiratory region in different mice at the same or different doses (Fig. 6a).

To specifically assess the feasibility of tracking bioluminescent IAV at various respiratory sites, determination of normalized bioluminescence flux from the nasal turbinates, trachea, lungs, and whole body showed that photon flux was detected above baseline in infected vs. mock-infected mice at all anatomical respiratory regions and doses of infection (Fig. 6b). Importantly, this included infection at a sublethal dose of 2 PFU that showed no visible bioluminescence upon imaging (Fig. 6a). With sublethal doses of IAV (2 and 20 PFU), body weight began to decrease during the prodromal period as early as 4 days after infection but continued to recover during convalescence (Fig. 6c). At 2 PFU, relative flux signals were observed at 2 days after infection and didn’t return to baseline until 9 days post infection, long after body weight had recovered. At 20 PFU, relative flux was also observed as early as 2 days post infection and increased between days 4 and 7 as body weight decreased. Body weight began to increase after 7 days while the relative flux decreased. At 200 PFU, body weight decreased until 9 days after infection. The relative flux at the lung did not increase above 10, but relative flux at the nasal turbinates and trachea increased to > 10 and rapidly decreased to 1 by 9 days after infection, at the beginning of convalescence. At 2000 PFU, body weight began to decrease during the prodrome period at 1 day post infection. Further, it decreased substantially during the invasion period until 9 days after infection. Mice began to die when the relative flux at the lung was greatest from 6 to 9 days after infection. Interestingly, the relative flux did not increase above 10 at any anatomical respiratory regions with sublethal doses (2 and 20 PFU) but did increase to > 10 in at least two anatomical respiratory regions with lethal doses (200 and 2000 PFU), suggesting that relative flux could be used to define the severity of IAV infection and ultimately the prognosis.

To compare the spatiotemporal replication of PB2-C-NanoLuc IAV as a function of infectious dose, relative flux from the nasal turbinates, trachea, and lung was plotted for each dose (Supplementary Fig. 3). Relative flux from lethal dose groups increased to > 10 at the nasal turbinates (200 and 2000 PFU), trachea (200 PFU), and lung (2000 PFU). Given that the relative flux from nasal turbinates and trachea in the 200 PFU group was > 10 for only 1 day, and the flux from nasal turbinates and lung in the 2000 PFU group was > 10 for 4 days, it seems that the duration of increased relative flux > 10 is inversely correlated with survival. Although the relative flux from the whole body (head and torso) had similar patterns to flux from the lungs (Fig. 6b,c), the application of relative flux from each respiratory site is of benefit to explain the dynamic replication of IAV throughout the respiratory tract. For example, while the lung flux at 20 and 200 PFU was < 10, the high flux in the nasal turbinates and trachea (> 10 relative flux) at 200 PFU distinguished the severity of infection from infection with 20 PFU (Supplementary Fig. 3).

### Infectious virus titers from the respiratory tract correlated with in vivo and in vitro bioluminescence

**Figure 7:**
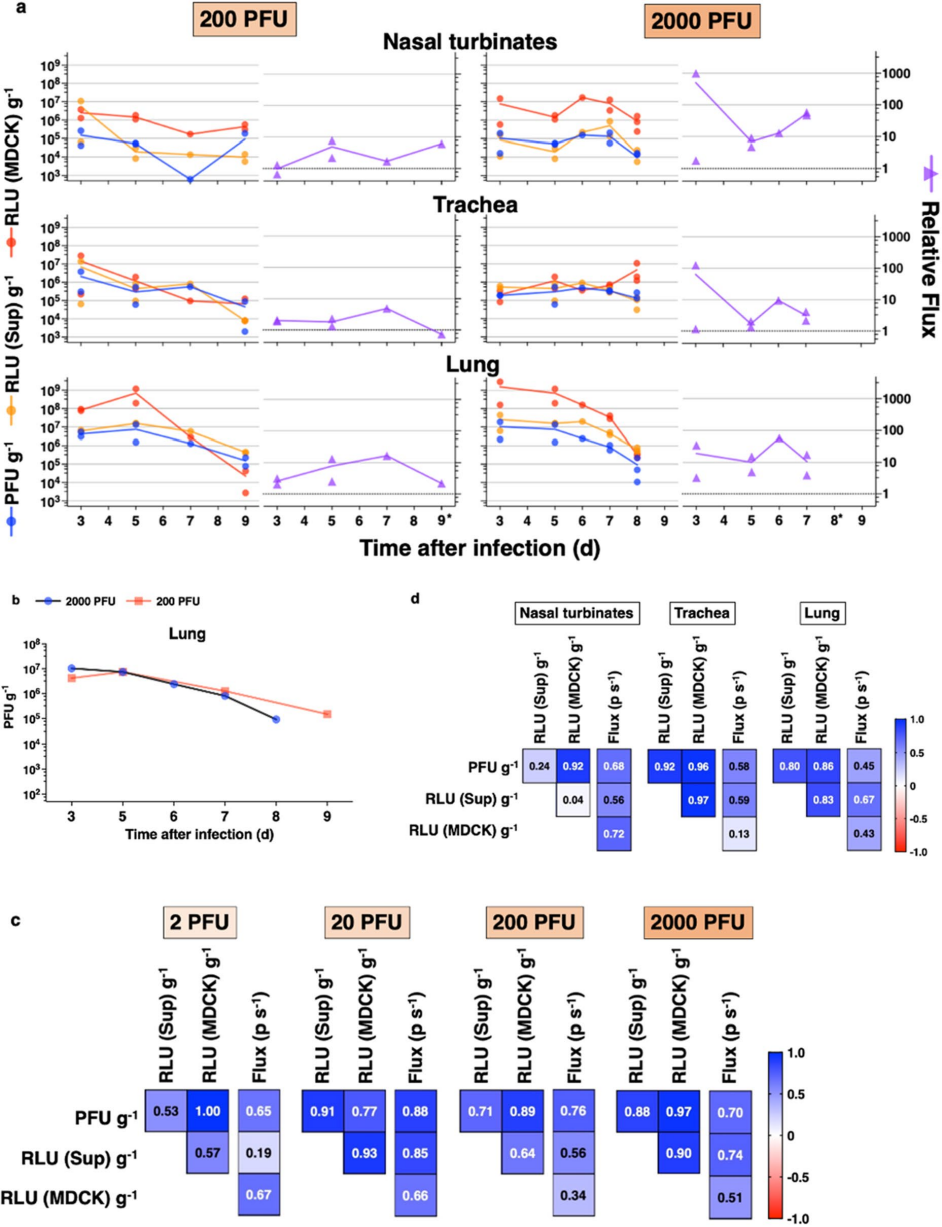
Correlation of in vitro measurements from tissue homogenates and in vivo bioluminescence from live mice. (**a**) In vitro measurements from plaque assay (PFU g^−1^), in vitro luciferase assay (RLU (Sup) g^−1^ and RLU (MDCK) g^−1^), and in vivo bioluminescence (relative flux) for infection with 200 and 2000 PFU. After infection with 200 or 2000 PFU, nasal turbinates, trachea, and lung were collected from 2 mice each at 3 and 5 days after infection. Additional tissues were harvested from 3 mice (200 PFU dose) and 6 mice (2000 PFU dose) which were moribund after terminal IVIS imaging. Mean values are connected by a line. *Mice that had no in vivo imaging because of severe dehydration and tachypnea. (**b**) Mean infectious viral titers (PFU g^−1^) from mouse lung homogenates vs time after infection with IAV at 200 or 2000 PFU. (**c,d**) Pearson correlation coefficient matrix for plaque assay (PFU g^−1^) and in vitro luciferase assay for supernatant (RLU (Sup) g^−1^) and MDCK cells (RLU (MDCK) g^−1^), and nonparametric Spearman correlation coefficient for flux (p s^−1^) from 63 measurements in different infectious dose groups and respiratory organs. *p* values are shown in Supplementary Table 1. Number of data for comparison: 2 PFU, 12 data; 20 PFU, 12 data; 200 PFU, 18 data; 2000 PFU, 21 data; nasal turbinates, trachea, and lung, each 21 mice. *PFU* plaque forming units, *RLU* relative luciferase units, *Sup* supernatant.

To evaluate the correlation between infectious titers from the respiratory tract vs. photon flux in vivo and luciferase signals in vitro, we measured MDCK cell plaque formation and luciferase activity in tissue homogenates from the nasal turbinates, trachea, and lung (Fig. 7a). Bioluminescent IAV in the lung peaked at 10^6^–10^7^ PFU g^−1^ at 3 to 5 days after infection with 200, or 2000 PFU. While bioluminescence imaging and flux measurement from the nasal turbinates, trachea, and lung indicated replication sites of IAV and corresponded to body weight changes and prognosis (Fig. 6c), infectious titers from lung tissue did not distinguish the 200 vs. 2000 PFU IAV doses and lethal outcomes (Fig. 7b). The 3 variables for plaque assay (PFU g^−1^), luciferase assay of supernatant [relative luciferase units (RLU) (Sup) g^−1^], and luciferase assay of MDCK cells [RLU (MDCK) g^−1^] strongly correlated with each other throughout the various dose groups (Fig. 7c), the trachea, and lung (Fig. 7d), but not the nasal turbinates where RLU (Sup) g^−1^ correlated poorly with PFU g^−1^ and RLU (MDCK) g^−1^. Since PFU g^−1^ correlated strongly with RLU (MDCK) g^−1^ in the nasal turbinates (Pearson correlation coefficient, 0.92), the poor correlation in the nasal turbinates was most likely due to measurement of RLU (Sup) g^−1^. Homogenates of nasal turbinates are composed primarily of bone tissue, which may cause bias in RLU (Sup) normalized by the weight of nasal turbinates. In contrast, the flux measurement from the different dose groups and respiratory tract correlated moderately with PFU g^−1^, RLU (Sup) g^−1^, and RLU (MDCK) g^−1^ (Fig. 7c,d).

**Figure 8:**
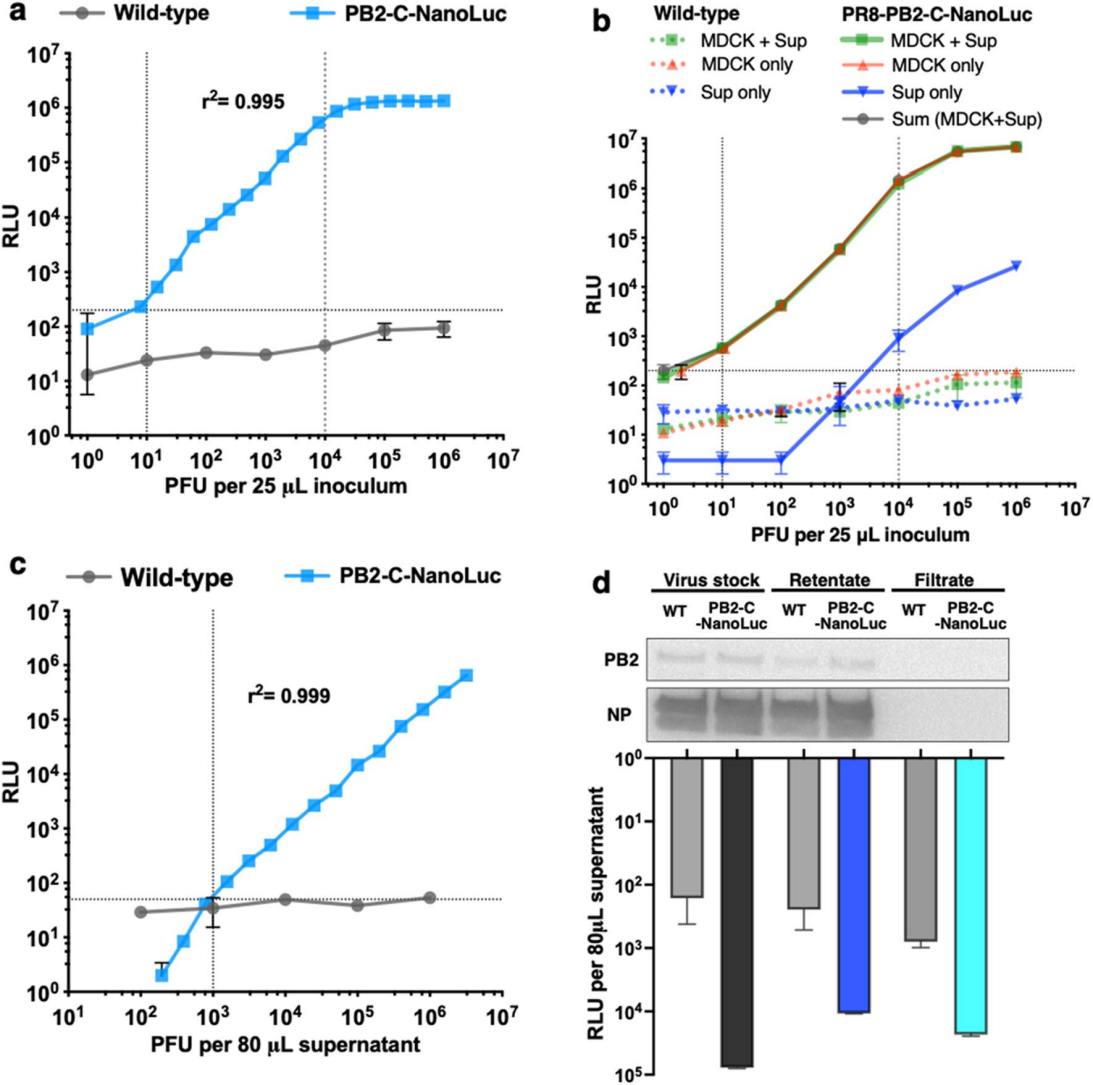
Linear detection range of PB2-C-NanoLuc IAV. Results reported as mean ± SD relative luciferase units (RLU) for triplicate measurements. (**a**) Linear detection range of PB2-C NanoLuc IAV in MDCK cells. Serially diluted samples (25 μL each) of wild-type or PB2-C-NanoLuc IAV were inoculated onto MDCK cells in white 96-well plates. At 12 h after inoculation, equal volume of luciferase substrate (80 μL) was added and luminescence was measured from MDCK cells and supernatant combined. There was strong correlation between measured luciferase activity vs infectious IAV titer from 10^1^ to 10^4^ PFU per 25 μL of inoculum (r^2^, 0.995; *p* < 0.0001). (**b**) Comparison of luciferase activity between MDCK cells and supernatants of MDCK cultures infected with PB2-C-NanoLuc IAV. To determine the source of the luciferase activity in the infected MDCK cultures, we separated MDCK cells and culture supernatants and measured the luciferase activity separately. At 12 h after inoculation of wild-type or PB2-C-NanoLuc IAV onto MDCK cells, luciferase activity was measured from MDCK cells and supernatant combined (squares; same method as in (**a**)), MDCK cells only (triangles; replacing with 80 μL PBS before adding 80 μL substrate), and supernatant only (upside-down triangles; adding 80 μL substrate); sum of data from MDCK only and supernatant only (circles). Within the linear range of detection (10^1^–10^4^ PFU per 25 μL inoculum), the luciferase activity from MDCK cells and supernatants combined was most comparable to that from MDCK cells alone. (**c**) Linear detection range of PB2-C NanoLuc IAV in supernatants. Luciferase activity of serially diluted 80 μL of wild-type or PB2-C-NanoLuc IAV was measured without infection to MDCK cells (same method of supernatant only as in (**b**)). Luciferase activity correlated with IAV PFU concentration linearly at PFU > 10^3^ per 80 μL supernatant (r^2^, 0.999; *p* < 0.0001). (**d**) Stocks of wild-type and PB2-C-NanoLuc IAV were filtered (100 kDa cutoff), and the retentates and filtrates were adjusted to the original volume (1 mL) and analyzed via luciferase assay and Western blot for PB2 and nucleoprotein (NP) as a marker for the presence of IAV. Cropped blots are displayed. Full-length blots are presented in Supplementary Fig. 9c.

To determine the linear detection range of PB2-C-NanoLuc IAV in MDCK cells, serially diluted wild-type or PB2-C-NanoLuc IAV were used to infect MDCK cells, and luciferase activity was measured at 12 h after infection. The background noise level was like luciferase activity from wild-type IAV, and there was a strong correlation between luciferase activity from PB2-C-NanoLuc IAV-infected MDCK cells vs. infectious viruses in the inoculum (Fig. 8a). This strong correlation was also observed when trypsin was omitted from the culture medium (Supplementary Fig. 4), supporting that the linear detection range of luciferase assay results were not affected by the presence of trypsin in culture media. Within the linear range of detection, the luciferase activity from MDCK cells and supernatant combined was most comparable to that from MDCK cells alone (Fig. 8b). Supernatants alone also had luciferase activity, consistent with the presence of NanoLuc protein, however, this activity was 1000-fold less than the activity from MDCK cells suggesting that luciferase activity was primarily associated with the cells (Fig. 8b).

Luciferase activity from PB2-C-NanoLuc IAV stocks alone, which were collected from the supernatant of infected MDCK cells, was detected linearly at a concentration of greater than 10^3^ PFU per 80 μL (Fig. 8c). To elucidate where the luciferase activity was coming from, centrifugal filtration of wild-type and PB2-C-NanoLuc IAV stocks was employed followed by Western blot of the stock vs. the retentate vs. the filtrate. Western blot showed that most of the IAV remained in retentate after filtration (Fig. 8d). However, luciferase activity was notable in both the filtrate and the retentate (Fig. 8d), suggesting that free NanoLuc protein was released into supernatants during the lytic replication of IAV when producing stocks. After 2 additional rounds of centrifugal filtration in separate experiments, IAV was isolated to the retentate and remained associated with some luciferase activity, suggesting that the NanoLuc protein was associated with IAV virions in some way (Supplementary Fig. 5a,b). Given that most of the bioluminescence signal arose from NanoLuc protein associated with infected cells as opposed to free or IAV-associated NanoLuc proteins (Fig. 8b,d), flux was not strongly correlated with PFU g^−1^ from various respiratory regions, which measured the amount of infectious IAVs present in the airway (Fig. 7d).

**Figure 9:**
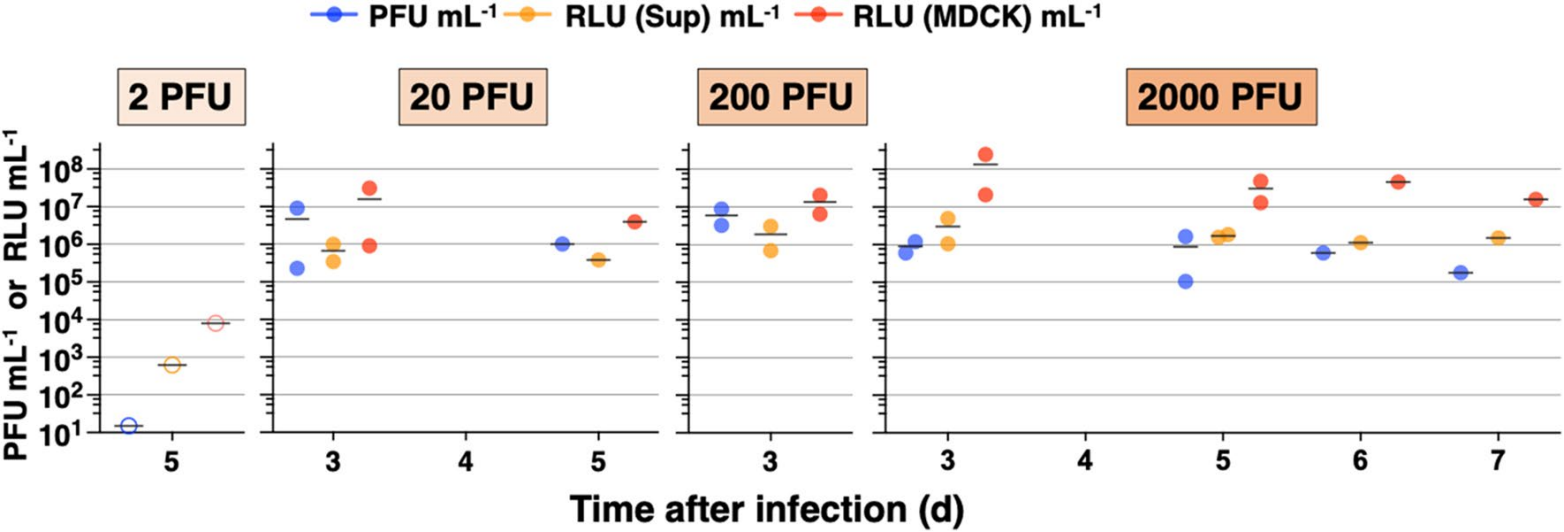
In vitro measurements on BALF samples to compare plaque vs luciferase assay. BALF was collected during the terminal procedure from 1 mouse or 2 mice at each inoculation dose (2, 20, 200, or 2000 PFU) of IAV and evaluated with in vitro plaque assay **(**PFU mL^−1^) and luciferase assay on supernatant (RLU (Sup) g^−1^), and MDCK cells (RLU (MDCK) g^−1^). Mean, black line. Hollow circles indicate measurements that were below the limit of detection of BALF samples for plaque assay (15 PFU mL^−1^) or luciferase assay on supernatant (625 RLU (Sup) mL^−1^) or MDCK cells (8000 RLU (MDCK) mL^−1^). *PFU* plaque forming units, *RLU* relative luciferase units, *Sup* supernatant.

To determine whether bioluminescent IAV released into the airway may be detected and quantified in real-time with the in vitro luciferase assay, bronchoalveolar lavage fluid (BALF) was collected during the terminal procedure and evaluated (Fig. 9). The BALF samples from mice infected with 20, 200, and 2000 PFU showed poor correlation between PFU and RLU (Sup) (Pearson’s r, 0.14) or between PFU and RLU (MDCK) (Pearson’s r, − 0.08). There were no PFU or RLU measured with infection at 2 PFU supporting that no infectious virus was present in the BALF of mice infected at this level. Real-time titration of IAV in BALF by luciferase assay on supernatant would save time over the plaque assay in MDCK cells which may require 12 h to 2 days of assay running time but should be applied only as a quick reference for viral shedding since the plaque assay is more quantitative.

## Discussion

The present study showed that PB2-C-NanoLuc IAV was tracked in real-time, even in mice infected with sublethal IAV doses. PB2-C-NanoLuc IAV maintained efficient replicative ability in MDCK cells, and virulence in mice was not compromised. The combination of in vivo imaging and photon flux measurement from the various anatomical regions of the respiratory tract demonstrated sensitive tracking of bioluminescent IAVs in mice over time, and furthermore bioluminescent signals from animals challenged at different doses explicitly corresponded to body weight changes and prognosis. In contrast, traditional infectious titers collected from lungs (Fig. 7b) did not distinguish the various dose levels and lethal outcomes. Together, these data support that real-time imaging of IAV replication will serve as an excellent research tool to aid in the development of vaccine and antiviral therapy where body weight changes alone had been traditionally used for monitoring clinical progress.

As opposed to previous studies attempting to develop bioluminescent IAVs expressing NanoLuc, the self-cleaving T2A peptide sequence was used in this study due to its efficient expression and cleavage of the second gene in a bicistronic construct. The T2A peptide sequence allowed correct subcellular localization of both proteins expressed from the bicistronic message, compared to the P2A peptide sequence from Porcine teschovirus-1^20–22^. In other studies, the GLuc reporter and the F2A self-cleavage sequence of foot-and-mouth disease virus were inserted into the C-terminus of PB2 to generate PR8-Gluc IAV; however, the virulence of that virus was significantly attenuated compared with wild-type PR8 virus^3^. This attenuation was likely due to inefficient cleavage of the F2A sequence in the PB2-GLuc polyprotein that affected viral replication^29^ and further supported the use of T2A as an alternative in these studies.

Importantly, the PB2-C-NanoLuc IAV was passaged stably in MDCK cells at least through 3 passages when point mutations were noted in the HA gene sequence. Point mutation in the NanoLuc gene of PB2-C-NanoLuc IAV was not observed until the sixth passage in MDCK cells, suggesting that marginal selective pressure was present, albeit weak. Therefore, it was concluded that all bioluminescent IAVs should be used within 3 passages from recovery in MDCK cells to avoid any unwanted mutation.

While previously reported bioluminescent IAVs with NanoLuc in the PA-C segment were rescued in the background of A/California/04/2019 and A/WSN/33^4–6^, we were unable to recover PA-C-NanoLuc IAV in the background of A/PR8/34. When the NanoLuc gene was replaced with a small peptide (HiBiT) to generate PA-C-HiBiT, the recombinant IAV replicated efficiently, suggesting that the length of the inserted sequence in the C terminus of PA affects the viability of IAV replication in MDCK cells. We speculated that changes in the length of the PA segment could have affected the results, including the repeated packaging signal sequence (60 nt for our study vs. 50 nt for others) and self-cleaving peptide (63 nt in T2A for our study vs. 66 nt in P2A for others). In addition, the bioluminescent IAVs in these previous studies were propagated in chicken embryonated eggs which often results in the acquisition of adaptive changes^30,31^. We purposefully chose propagation in MDCK cells for that reason.

We also demonstrated that use of the small HiBiT peptide tag as opposed to larger reporters was beneficial in generating bioluminescent IAVs through successful recovery of IAVs tagged with HiBiT in both the N- and C-terminus of the PB2 and PA gene segments. In each case, the HiBiT tag was stably maintained through passage, and efficiently released via T2A self-cleavage to enable a complex with LgBiT and emission of bioluminescent signals in infected MDCK cells. Only the PB2-N-HiBiT and PB2-C-HiBiT IAVs replicated as efficiently as wild-type IAV in cells (Fig. 2b,c), providing further support that the PB2 gene segment of PR8 IAV, as opposed to the PA gene segment, is more tolerant to insertions. PB2-N-HiBiT and PB2-C-HiBiT IAVs also infected mice similarly to wild-type IAV (Fig. 4b); however, we were unable to image PB2-N-HiBiT and PB2-C-HiBiT IAVs in mice likely due to insufficient delivery of LgBiT protein to infected cells in the respiratory tract. To overcome this obstacle, HiBiT tagged IAVs could be administered to LgBiT-expressing transgenic mice or regular animals that express LgBiT via viral vectors. Alternatively, secretion or targeting self-cleaved HiBiT peptide via T2A sequence to the plasma membrane by signal peptide sequence may bypass the difficulty of delivering LgBiT protein to cells in live animals. Regardless, current data generated here support use of PB2-N-HiBiT and PB2-C-HiBiT IAVs to track infection in tissue culture cells.

For mice infected with PB2-C-NanoLuc IAV, we showed that the luciferase assay was able to detect free and virus-associated NanoLuc protein in the supernatant from tissue homogenates and BALF. However, the real-time luciferase assay of supernatant from BALF is only beneficial as a quick reference for monitoring viral shedding in transmission studies, for example, since plaque titration in MDCK cells is more quantitative. Bioluminescence from cell-free NanoLuc proteins was almost negligible compared with the total bioluminescence measured from live animals suggesting that NanoLuc protein remains predominantly associated with infected cells. Consequently, the bioluminescence signal (photon flux) was only modestly correlated with infectious titer (PFU). PFU g^−1^ after infection was generally higher at 3 than 5 days after infection (Fig. 7a), while photon flux was consistently higher at 5–6 days after infection (Fig. 6c). Previous studies showed similar discrepancies between PFU g^−1^ and flux^4^. As the most intense bioluminescence signal from NanoLuc protein originated from cells infected with bioluminescent IAVs, the expression of NanoLuc during infection may necessitate a slight delay. Lastly, we demonstrated that real-time tracking in live mice showed the intensity and duration of flux, which were indicative of the extent and duration of airway infection with IAV, provided a better prediction of disease progression and prognosis than traditional infectious titers in respiratory organs.

For some of the images shown in Fig. 6a, we observed different infection patterns from each mouse in the same infectious dose group even though our IVIS imaging experiments were performed using the same infection and imaging procedure. Specifically, all mice were infected intranasally after isoflurane anesthesia using a SomnoSuite (Kent Scientific) at a consistent isoflurane concentration in the anesthetic chamber. Each animal, however, reacted slightly differently to the isoflurane. For example, some animals fell asleep more deeply and more rapidly. IAV in the inoculum is administered intranasally while animal’s inhale. Thus, we speculate that slightly different anesthetic planes and associated breathing rates could result in slightly different infection patterns. The various infection patterns observed in our IVIS imaging agree with natural infection in humans or animals. However, when comparing total flux among different infectious doses, overall infection patterns were distinct per infectious dose, as shown in Fig. 6b,c.

PB2-C-NanoLuc IAV infection of tissues other than the respiratory tract was not noted in these IVIS imaging studies, though infectious wild-type PR8 can be recovered from the brain of infected mice (unpublished data). We speculate that PB2-C-NanoLuc IAV would also spread to the brain at high doses and previous study have shown that furimazine, NanoLuc substrate, can reach the brain across the blood–brain barrier^32^. Thus it is likely that the blue emission (maximum wavelength, 460 nm) of NanoLuc bioluminescence may be suboptimal for in vivo imaging of deep tissues^33^. Further study is justified with PB2-C-NanoLuc IAV using improved substrates such as modified furimazine with red-shifted emission^34^ that may improve tracking sensitivity in deeper tissues. Alternatively, in vivo imaging of deep tissues may be improved by enhancing bioluminescent intensity by using substrates which improved the solubility of furimazine^35^. Recent advancements in firefly luciferase (FLuc) resulted in approximately 13 times brighter red-shifted emission by substituting 28 amino acids in FLuc to generate AkaLuc^36^. AkaLuc allowed deep tissue imaging up to the single-cell level of bioluminescence in living animals and could be used as an alternative to NanoLuc in bioluminescent IAV. However, the relatively large size of AkaLuc (1653 nt), may restrain IAV replication when inserted into the PB2-C terminus.

Lastly, both BALB/c and C57BL/6 mice are typically used for pathogenicity studies in IAV infection. However, C57BL/6J mice are more susceptible to select IAV strains such as 2009 pandemic H1N1 IAVs^37^. As most transgenic mice have been developed from C57BL/6 mice, the similar pathogenicity of wild-type and PB2-C-NanoLuc PR8 IAVs in C57BL/6 mice may enable functional evaluation of host proteins in live animals. For example, PB2-627K adaptation in avian IAVs is important to take advantage of mammalian ANP32A protein for viral polymerase assembly^38–40^, and our real-time tracking IAV may be used to evaluate the mechanisms of ANP32A adaptation in live mice. Also, with tracking sensitivity for the heterogeneous replication of IAV in live animals, PB2-C-NanoLuc PR8 IAV may enable the evaluation of immune response heterogeneity in the dirty mouse model, which was proposed to refine misleading vaccine results from inbred mice^41–43^.

## Methods

### Plasmids and cells

The A/PR8/34 reverse genetics plasmids were provided by Yoshihiro Kawaoka (University of Wisconsin-Madison), including 8 pHH21 plasmids (pHH21-PB2, PB1, PA, NP, HA, NA, M, and NS) for production of viral RNAs and 4 pCAGGS plasmids (pCAGGS-PB2, PB1, PA, and NP) for expression of viral polymerase complex and NP^23^. Reporter genes NanoLuc, moxGFP, and iCre were amplified from the plasmids pUAS-NanoLuc, pmoxGFP and paavCAG-iCre, respectively, provided by Robert Campbell (Addgene plasmid #87696; http://n2t.net/addgene:87696; RRID: Addgene_87696)^44^, Erik Snapp (Addgene plasmid #68070; http://n2t.net/addgene:68070; RRID: Addgene_68070)^23^, and Jinhyun Kim (Addgene plasmid #51904; http://n2t.net/addgene:51904; RRID: Addgene_51904)^24^.

293T cells (ATCC, CRL-11268) were grown in Dulbecco Modified Eagle Medium (DMEM) (ATCC) supplemented with 10% fetal bovine serum (FBS) (ATCC) and 1% penicillin–streptomycin (Gibco). MDCK cells (London Line, FR-58) were obtained (International Reagent Resource, Influenza Division, WHO Collaborating Center for Surveillance, Epidemiology and Control of Influenza, Centers for Disease Control and Prevention) and grown in Eagle Minimum Essential Medium (EMEM) containing 1% nonessential amino acids (Gibco), 1% GlutaMAX-1(Gibco), 1 mM sodium pyruvate (Gibco), 1.5 g L^−1^ sodium bicarbonate (Gibco), and 10% FBS. MDCK cells were gradually adapted to grow in serum-free medium (OptiPRO SFM, Gibco) with 2% supplement (GlutaMAX-1) for 5 passages over 1 month. Since PR8 replication in MDCK cells did not differ between maintenance media [EMEM containing 1% nonessential amino acids, 1% GlutaMAX-1, 1 mM sodium pyruvate, 1.5 g L^−1^ sodium bicarbonate, 0.1% bovine serum albumin (BSA), and 0.5 μg mL^−1^ trypsin treated with N-tosyl-l-phenylalanine chloromethyl ketone (TPCK-trypsin)] vs serum-free medium (Supplementary Fig. 6), we used serum-free medium for the entire study to avoid FBS washing steps before inoculation. All cells were grown in an incubator with 5% carbon dioxide and limited to 20 passages.

### Generation of bioluminescent IAVs

The PB2 and PA fusions with NanoLuc and HiBiT were created in the pHH21 plasmid (In-Fusion Cloning, Takara Bio) (Fig. 1a,b). Four DNA fragments (3′-PB2-, 5′-PB2-, 3′-PA-, and 5′-PA-DNA) were synthesized (GENEWIZ). The other parts of the native sequences from PB2 or PA were amplified by polymerase chain reaction (PCR) using the templates of pHH21-PB2 or pHH21-PA, and 2 fragments from each 3′- and 5′-side were cloned into the pHH21 plasmid (In-Fusion Cloning, Takara Bio). The gene segments containing NanoLuc, moxGFP and iCre (Supplementary Fig. 1), were generated by replacing the HiBiT tag sequence with the reporter genes. Plasmid constructions were designed using software (SnapGene, version 5.1), and all constructed plasmids were sequenced to ensure that there were no unwanted mutations (GENEWIZ).

Reporter IAVs were rescued using reverse genetics methods by transfection with a 12 plasmid system of IAV A/PR8/34 (H1N1) strain in 293T cells as described previously (Supplementary Fig. 7)^23^. For reporter IAVs that could not be rescued on the first attempt, we made 2 additional rescue attempts by modifying the regular reverse genetics procedure with optimized viral rescue conditions including optimized amounts of pCAGGS plasmid, pHH21 plasmid, transfection reagent, incubation time, and coculture time in 293T and MDCK cells after transfection (Supplementary Fig. 7a–e).

Serial viral passage in MDCK cells for stability experiments were performed (MOI, 0.001). Confluent MDCK cells in T75 flasks were infected with bioluminescent PR8 IAVs for 1 h at 37 °C. The inoculum was removed and MDCK cells were washed twice with PBS. Fresh growth medium (OptiPRO SFM supplemented with 2% GlutaMAX-1 and 0.5 μg mL^−1^ of TPCK-treated trypsin; 10 mL) was added, and the infected MDCK cells were incubated at 37 °C for 3 days. Harvested IAVs were filtered with a 0.45 μm polyethersulfone filter (Fisherbrand), divided into aliquots, and stored at − 80 °C. All stocks of reporter IAVs were made in MDCK cells within 3 passages of growth for 3 days at 33 °C and sequenced after PCR amplification of all 8 segments to ensure that there were no unwanted mutations (GENEWIZ).

### Multi-step growth curve

Confluent MDCK cells in T25 flasks were infected with bioluminescent PR8 IAVs or wild-type PR8 (MOI, 0.001) for 1 h at 37 °C. The inoculum was removed and the cells were washed twice with PBS. Fresh medium (5 mL) containing TPCK-trypsin (0.5 μg mL^−1^) was added, and the infected MDCK cells were incubated at 37 °C or 33 °C for 4 days. At various times, supernatant (0.3 mL) was collected and replaced with fresh media (0.3 mL). Collected supernatants were cleared by centrifugation (3000 rpm for 5 min) and stored at − 80 °C until viral titration was done by plaque assay.

### Plaque assay

Plaque assay was done in MDCK cells in 24- or 48-well plates. At 1 h after inoculation at 37 °C, infected MDCK cells were overlayed with a 2: 1 mixture of growth medium and microcrystalline cellulose (3.6% Avicel, RC-591, DuPont) including TPCK-treated trypsin (0.5 μg mL^−1^) and incubated at 37 °C. At 3 days post infection, MDCK cells were fixed with 10% buffered formalin for 1 h. After removing the overlay, MDCK cells were permeabilized with PBS containing 0.5% Triton X-100 and 20 mM glycine for 20 min. The plates were washed three times with PBS containing 0.05% Tween 20 (PBST), and plaques were immunostained with a 1: 3000 dilution of anti-IAV-NP antibodies (MAB8257 and MAB8258, 1: 1 mixture, EMD Millipore) and peroxidase-labeled goat anti-mouse IgG (SeraCare). Immunostained plaques were treated with peroxidase substrate (TrueBlue, SeraCare) according to the manufacturer’s protocol and counted manually using an inverted light microscope (Laxco). The limit of detection in 24-well plates was 10 PFU mL^−1^ while that in 48-well plates was 15 PFU mL^−1^. Plaques from different IAVs were compared for size in 12-well plates by infecting cells at a MOI of 0.0002, incubation for 4 days under 0.8% agarose overlay, and staining with crystal violet (0.5%) (Fig. 2a).

Tissue collected from mice was homogenized in a bead homogenizer with a 1: 1 mixture of 1.4 mm and 2.8 mm ceramic beads in cold PBS containing 1% penicillin–streptomycin (Gibco) (trachea, 0.5 mL; nasal turbinates or lung, 1 mL). After homogenization (3 consecutive cycles, 30 s each) and chilling on ice (30 s), samples were centrifuged (5000 rpm) for 5 min at 4 °C. Cleared supernatants were divided into aliquots and stored at − 80 °C until titration. The limit of detection varied with tissue or fluid (nasal turbinates, 136 PFU g^−1^; trachea, 188 PFU g^−1^; lung, 65 PFU g^−1^; BALF, 15 PFU mL^−1^).

### In vitro luciferase assay

NanoLuc activity was evaluated in confluent MDCK cells in white 96-well plates that were infected with serially diluted IAVs (25 μL) for 1 h at 37 °C. After washing twice with PBS (100 μL per wash), growth medium (80 μL) containing TPCK-treated trypsin (0.5 μg mL^−1^) was replaced, and plates were incubated for 12 h at 37 °C. Luciferase substrate (Nano-Glo, Promega) was prepared according to the manufacturer’s protocol (1: 50 ratio of substrate and buffer), added to each well (80 μL per well), and incubated for 10 min at room temperature. After shaking (2 min), luminescence was measured with a plate reader (Synergy 2, BioTek) with 1 s integration time and auto gain. NanoLuc activity in the supernatant without MDCK cell infection was determined by mixing supernatant (80 μL) with luciferase substrate (Nano-Glo; 80 μL), incubation for 10 min at room temperature, and measurement of luminescence.

In vitro luminescence was measured from mouse tissue samples and BALF. The limit of detection varied with sample source in MDCK cells [nasal turbinates, 72,727 RLU (MDCK) g^−1^; trachea, 100,000 RLU (MDCK) g^−1^; lung, 34,783 RLU (MDCK) g^−1^; BALF, 8000 RLU (MDCK) mL^−1^] and supernatants [nasal turbinates, 5682 RLU (Sup) g^−1^; trachea, 7813 RLU (Sup) g^−1^; lung, 2717 RLU (Sup) g^−1^; BALF, 625 RLU (Sup) mL^−1^]. We determined HiBiT and LgBiT luciferase activity from IAV-infected MDCK cells by adding reagents (80 μL) prepared by mixing LgBiT protein, lytic substrate, and lytic buffer (1: 2: 100) (Nano-Glo HiBiT Lytic Detection System, Promega) and measuring luminescence according to the manufacturer’s protocol.

### Western blot

Whole-cell lysates from mock- or virus-infected (MOI, 0.001) MDCK cells were collected at 24 h after inoculation in radioimmunoprecipitation assay buffer containing protease inhibitors (Takara Bio), electrophoresed under reduced denaturing conditions (Bolt Bis–Tris Plus gel, Invitrogen), and transferred to a polyvinylidene difluoride membrane (Bio-Rad Laboratories). Membranes were blocked for 1 h with 5% dried skim milk (Research Products International) in PBST and incubated with polyclonal rabbit anti-PB2 (PA5-32220, Invitrogen), rabbit anti-PA (PA5-32223, Invitrogen), rabbit anti-NP (PA5-32242, Invitrogen), monoclonal mouse anti-NanoLuc luciferase (MAB10026, R&D systems), or mouse anti-β-actin (AM4302, Invitrogen) followed by horseradish peroxidase-conjugated anti-mouse (5220-0341, SeraCare) or antirabbit IgG (31460, Invitrogen). Specific proteins were detected using a chemiluminescent substrate (SuperSignal West Pico PLUS, Thermo Scientific) according to the manufacturer's recommendations and photographed (ChemiDoc XRS, Bio-Rad Laboratories).

IAVs in supernatants (1 mL) were separated with a filter unit (100 kDa cutoff) (Amicon Ultra-4 Centrifugal Filter Unit, Millipore Sigma) by centrifugation (swinging-bucket rotor; 4000×*g*) at 25 °C for 15 min. The retentate and filtrate were collected separately and adjusted to 1 mL with PBS. After aliquots were taken for luciferase determination and Western blot, 90% (volume/volume) of remaining retentates were processed for a second and third filtration. Western blot samples from supernatants (original stock, retentates, and filtrates) were prepared in sample buffer (4× Bolt LDS, Invitrogen) and sample reducing agent (10× Bolt, Invitrogen) and denatured for 10 min at 70 °C according to the manufacturer’s protocol. Whole cell lysates were processed similarly. Substrates were used to detect NP (SuperSignal West Pico PLUS Chemiluminescent Substrate, Thermo Scientific) or PB2 (SuperSignal West Femto Maximum Sensitivity Substrate, Thermo Scientific).

### Virulence in mice

Animal experiments and procedures were approved by the Institutional Animal Care and Use Committee (IACUC) at the University of South Alabama, carried out in accordance with relevant guidelines and regulations, and reported in accordance with the ARRIVE guidelines (https://arriveguidelines.org). Female C57BL/6 mice (age, 6–8 week) (Charles River) were maintained in the University of South Alabama vivarium. Mouse cohorts (randomly divided into 5 or 6 mice per group) for the virulence study and in vivo imaging were infected with wild-type or bioluminescent PR8 IAV (50 μL) intranasally under isoflurane anesthesia (SomnoSuite, Kent Scientific) at doses ranging from 0 PFU (mock infection) to 2000 PFU per mouse. All mice were monitored twice daily for 2 weeks to evaluate virulence including daily body weight and signs of morbidity and mortality. Mice that lost more than 20% of initial body weight were monitored three times daily including additional body weight measurement. Mice that lost more than 25% of initial body weight were humanely euthanized and the date recorded. The 50% mouse lethal dose (MLD_50_) was calculated by Reed and Muench method^45^. The natural history of influenza viral infection in animal was defined according to the change in body weight as a representative of clinical progression in a mouse including incubation, prodrome, invasion, and convalescence periods and recovered healthy condition (Fig. 6c).

### In vivo imaging

In vivo bioluminescence imaging was determined in mock-infected mice (PBS) or mice infected with bioluminescent PR8 IAVs using an in vivo imaging system (IVIS 200 Spectrum In Vivo Imaging System, PerkinElmer) with software (Living Image, version 4.7, PerkinElmer). For initial comparison of PB2-NanoLuc, PB2-N-HiBiT, and PB2-C-HiBiT IAV, each IAV (20,000 PFU) was inoculated into mice intranasally (two mice per virus), and imaged at 3 and 6 days post infection. Since the reference MLD_50_ for mice infected with PR8 IAV was ~ 100–300 PFU^7,46^, the dose of wild-type PR8 IAV was 2000 PFU per mouse. Mice were lightly anesthetized with isoflurane (SomnoSuite), and the hair was shaved from the neck and upper torso before 1st IVIS imaging. Fresh substrate (Nano-Glo, Promega) diluted 1: 10 with sterile PBS (total, 100 μL) was injected retro-orbitally^47^ with a 28-gauge, ½-inch needle. According to the IACUC approved protocol at the University of South Alabama, retro-orbital injection could be attempted only twice per eye per mouse after IAV infection, thus imaging was limited to 4 time points along with one additional injection for terminal imaging. For in vivo imaging of HiBiT-tagged IAV at 3 days post infection an injection mixture (total, 100 μL) was prepared with LgBiT protein, HiBiT lytic substrate, and sterile PBS at a ratio of 0.5: 1: 10, or 1: 1: 10. For 6 days post infection only the 1: 1: 10 ratio was used. This injection mixture was also delivered retro-orbitally as described above. Imaging was performed for PB2-C-NanoLuc within 30 s after substrate injection, and for HiBiT at 5 min after substrate injection, with emission filter open, field of view at 6.6 cm, f-number at 1, binning factor at small (4), and at an exposure time of 330 s (or an exposure time of 300 s for IVIS imaging in Fig. 4). Photon flux (number of photons per s) was determined from the nasal turbinates, trachea, and lung using software (Living Image, version 4.7, PerkinElmer), and relative flux was defined as the photon flux normalized by the mean flux from mock-infected mice.

### Statistics

Statistical analyses were performed with software (Prism, version 9.1.2, GraphPad, San Diego, CA). Comparisons were made using repeated-measures 2-way analysis of variance (ANOVA) with Greenhouse–Geisser correction and Dunnett multiple comparisons test for multi-step growth data, log-rank test for survival curves, Pearson correlation for plaque assay and luciferase assay on supernatants and MDCK cells, and nonparametric Spearman correlation for flux. Correlation coefficients and *p* values were displayed in a correlation matrix and table.

## Supplementary Information

Supplementary Information.
