# Supplementary material for: Real-time tracking of bioluminescent influenza A virus infection in mice
Source: Sci Rep. 2022 Feb 24;12:3152. doi: 10.1038/s41598-022-06667-w (PMC8873407; doi:10.1038/s41598-022-06667-w)
Supplement: Supplementary file 1 — Supplementary Information. [file 41598_2022_6667_MOESM1_ESM.pdf]

Supplementary information

Real-time tracking of bioluminescent influenza A virus infection in mice

Jin H. Kim[1, 2], Hannah Bryant[3], Edward Fiedler[1], TuAnh Cao[1] & Jonathan O. Rayner[1]

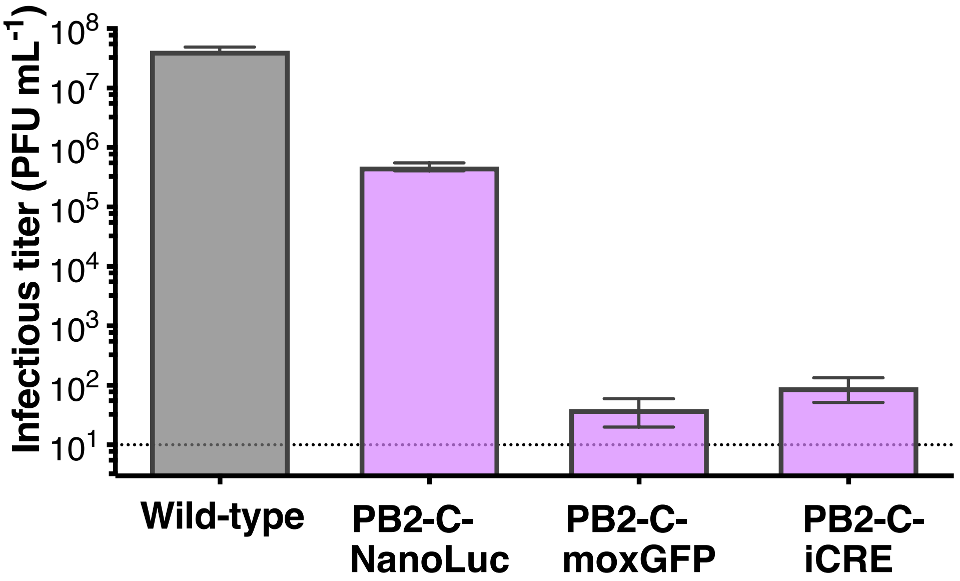

Supplementary Fig. 1. Generation of PR8 IAVs carrying moxGFP and iCRE at the PB2-C-terminus (PB2-C-moxGFP and PB2-C-iCRE). The longer reporter genes, monomeric oxidizing environment-optimized green fluorescent protein (moxGFP, 714 nt) and improved Causes recombinase (iCre, 1050 nt) containing nuclear localization sequence, were replaced for NanoLuc (510 nt) in the PB2-C-terminus, and rescued by reverse genetics in coculture of 293T and MDCK cells. The rescued IAVs were grown in MDCK cells at 33°C for 3 days, and infectious viral loads were titrated in MDCK cells by plaque assay. Data on PR8 wild-type and PB2-C-NanoLuc IAVs were imported from Fig. 1c and plotted for reference. PB2-C-moxGFP and PB2-C-iCRE IAVs had lower infectious titers than wild-type PR8 and PB2-C-NanoLuc. Data are reported as mean ± SD (N= 3 for all experiments).

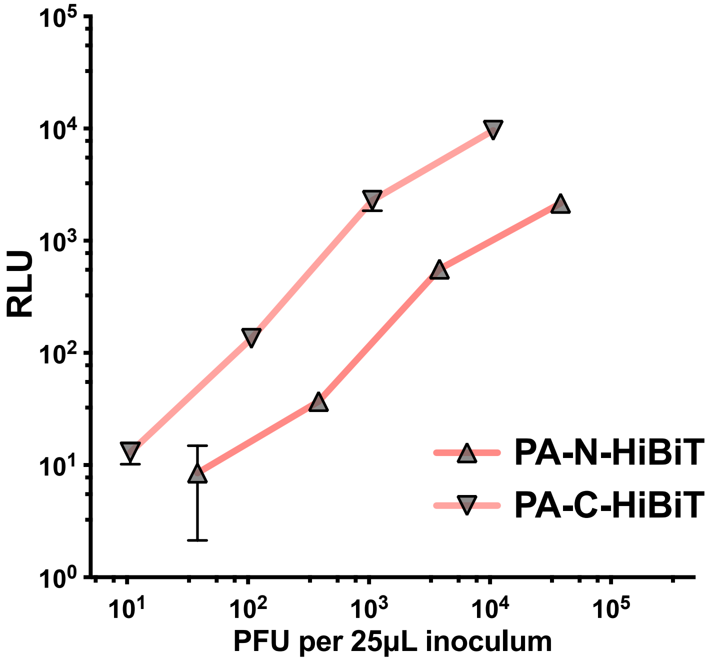

Supplementary Fig. 2. Luciferase assay in MDCK cells infected with PA-N-HiBiT and PA-C-HiBiT IAVs. Luciferase activity was determined in MDCK cells at 12 h after inoculation, indicating that the PA gene segment containing the HiBiT tag (PA-N-HiBiT or PA-C-HiBiT) was incorporated into virions. Data reported as mean ± SD (N = 3).

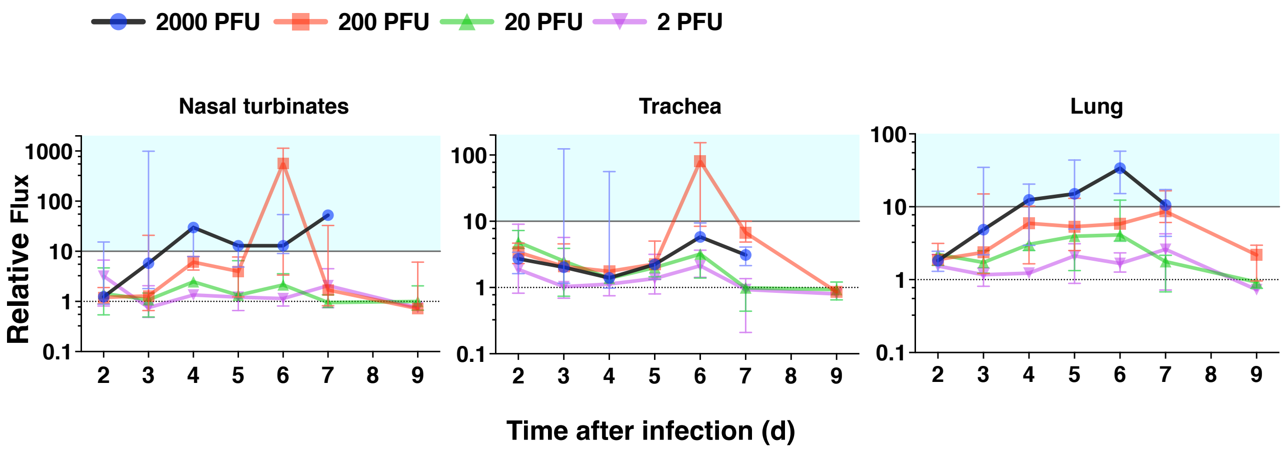

Supplementary Fig. 3. Spatiotemporal resolution of IAV replication kinetics in the mouse respiratory tract. Relative flux for the nasal turbinate, trachea, and lung at different IAV doses was replotted (median and 95% confidence interval). Relative flux > 10, light blue.

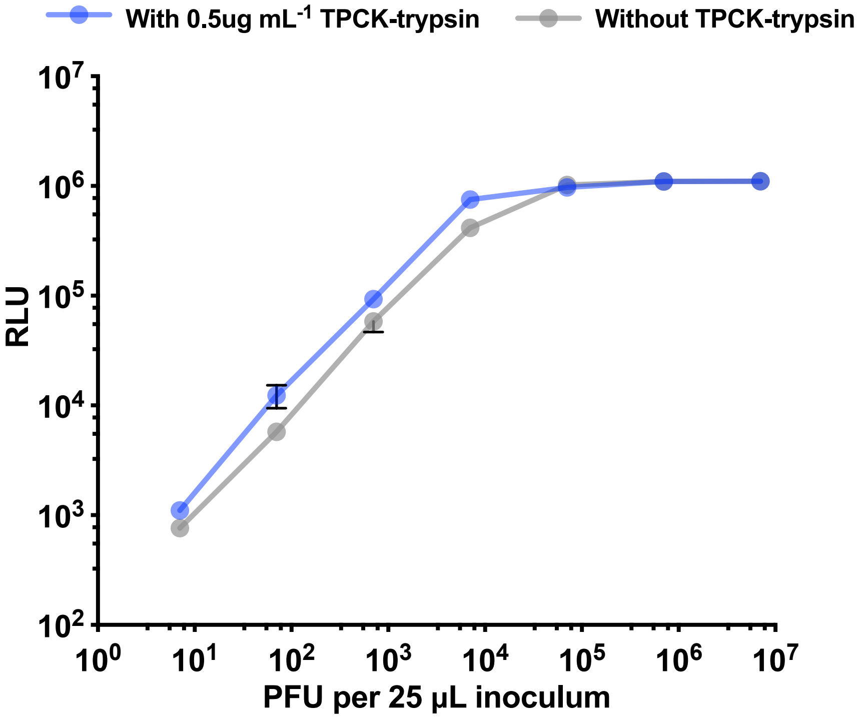

Supplementary Fig. 4. Luciferase assay in MDCK cells with and without trypsin in culture media. MDCK cells in a white 96-well plate were infected with PB2-C-NanoLuc and incubated at 37°C with or without 0.5 µg mL^{-1} of TPCK-treated trypsin. At 12 h after infection, luciferase substrate was added and luminescence was measured. Results reported as mean ± SD relative luciferase units (RLU) for triplicate measurements. Luciferase activity in TPCK-treated trypsin increased luciferase activity by 45% to 115% without affecting the linear range from 10^1 to 10^4 PFU per 25 µL of inoculum. Therefore, the presence of trypsin in the culture medium did not affect the linearity of PB2-C-NanoLuc luciferase activity in MDCK cells.

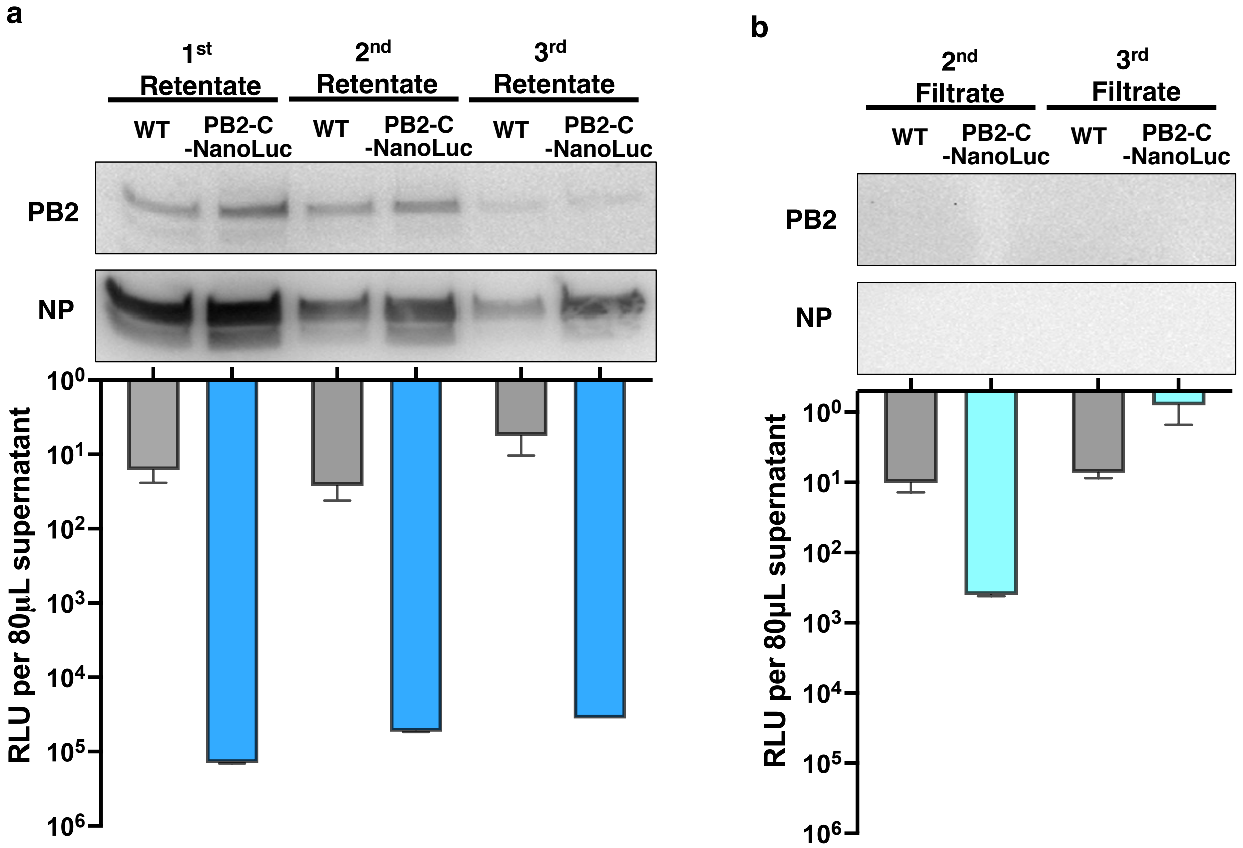

Supplementary Fig. 5. Luciferase assay and Western blot of wild-type and PB2-C-NanoLuc IAVs after repeated centrifugal filtration of viral stock solutions. After samples were taken from the retentate and filtrate for Western blot and luciferase assay, 90% (volume per volume) of the remaining retentates were subjected to a second and third round of centrifugal filtration. The volume of each retentate and filtrate was adjusted to the original stock volume (1 mL). The presence of wild-type and PB2-C-NanoLuc IAV was confirmed by Western blot with anti-PB2 and anti-NP antibody, and the presence of NanoLuc protein was confirmed by luciferase assay, demonstrating that NanoLuc protein remained associated with PB2-C-NanoLuc IAV. Cropped blots are displayed. Full-length blots are presented in Supplementary Fig. 9d. Luciferase data reported as Mean ± SD (N=3).

(**a**) Retentate from the first, second, and third rounds of centrifugal filtration showing the presence of PB2 and nucleoprotein (NP) in retentate of wild-type and PB2-C-NanoLuc IAV and presence of luciferase activity in retentate of PB2-C-NanoLuc above the background level from wild-type IAV.

(**b**) Filtrate from the second and third rounds of centrifugal filtration showing the absence of PB2 and NP in second and third filtrates and lower levels of luciferase activity in PB2-C-NanoLuc filtrate. After the third round of filtration, no further NanoLuc protein was filtered from retentates. This suggests that NanoLuc proteins in the third retentate were associated with virions.

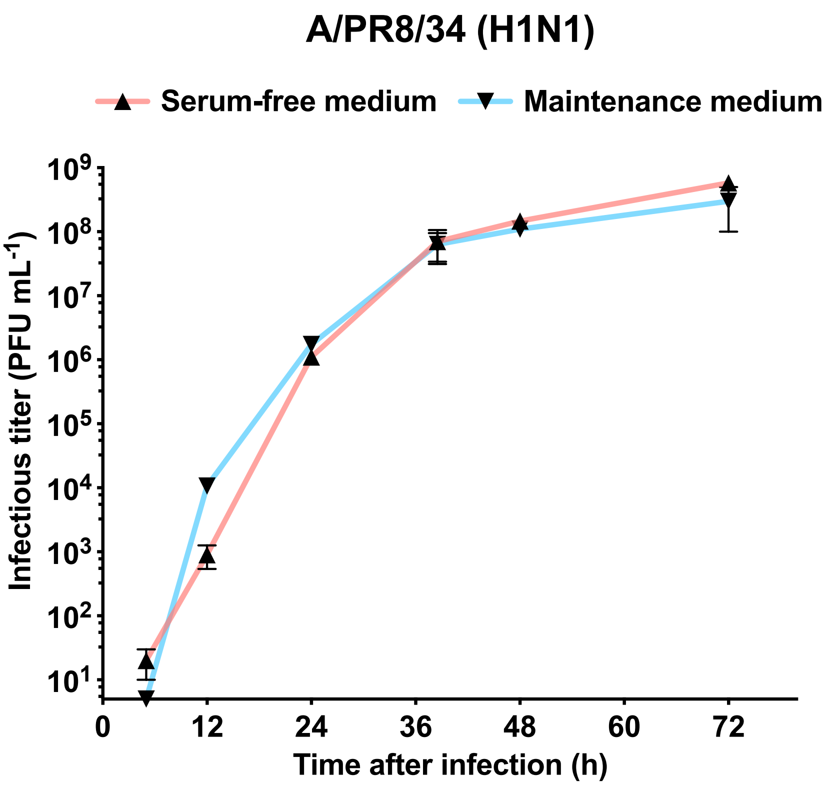

Supplementary Fig. 6. Growth properties of wild-type PR8 IAV in MDCK cells in maintenance medium or serum-free medium. Wild-type PR8 IAV was used to infect to MDCK cells in maintenance medium and serum-free medium (OptiPRO SFM containing 2% GlutaMAX-1, and 0.5 μg mL^{-1} TPCK-trypsin) at multiplicity of infection (MOI) of 0.01. Infected cells were incubated at 37°C for 4 days. The supernatants from each MDCK cell culture were collected at designated times and titrated by plaque assay on MDCK cells overlayed with maintenance media containing 0.8% agarose. Results reported as mean ± SD (N = 3).

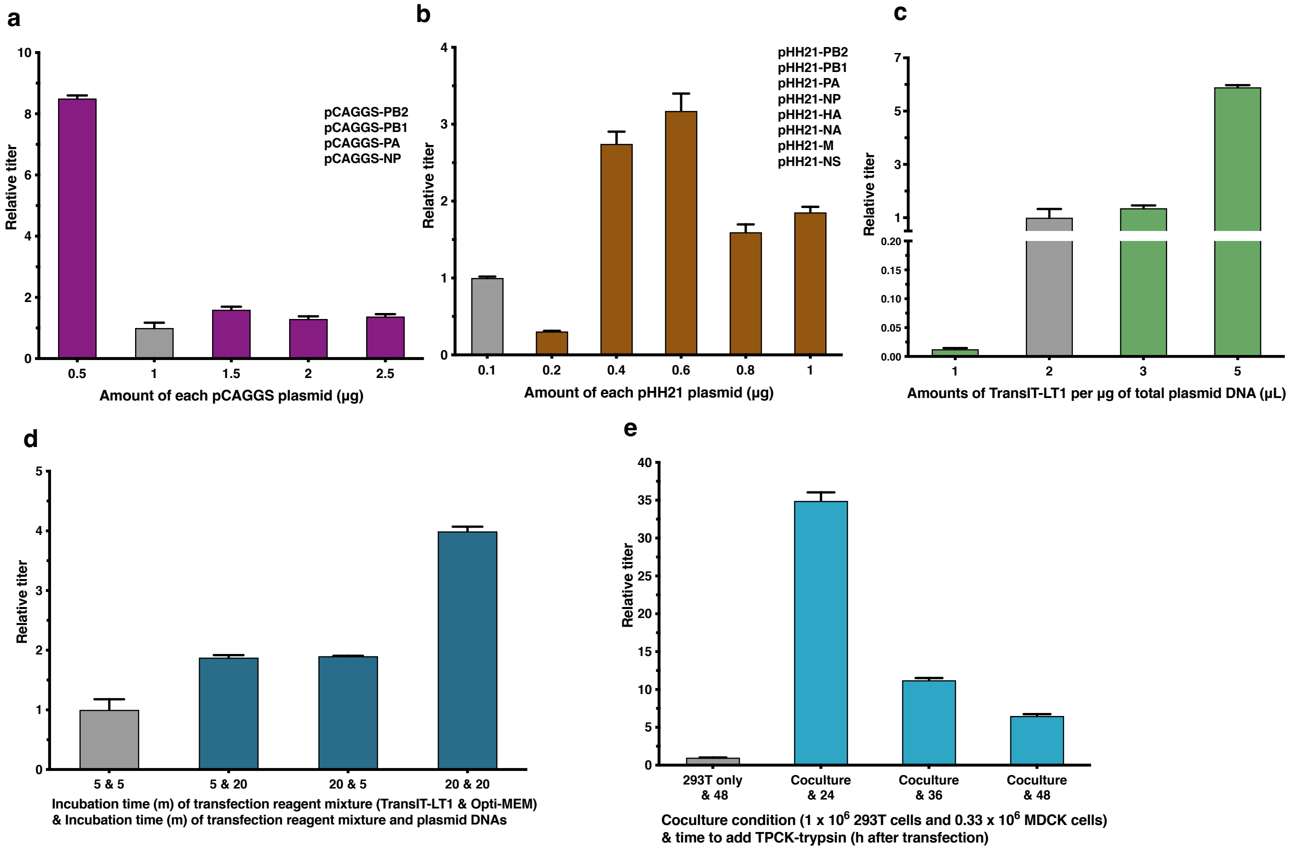

Supplementary Fig. 7. Optimization of reverse genetics for rescuing IAV. For the regular reverse genetics procedure in 293T cells (1 × 10^6 cells per 35-mm dish), transfection reagent (TransIT-LT1; 2 μL per 1 μg total plasmid DNA) was mixed with reduced-serum medium (Opti-MEM; 100 μL) for 5 min. Plasmid DNA for A/PR8/34 (1 μg of each pCAGGS-PB2, PB1, PA, and NP plasmid and 0.1 μg of each pHH21-PB2, PB1, PA, NP, HA, NA, M, and NS plasmid; total, 4.8 μg) was added to the transfection reagent mixture and incubated for 5 min, applied dropwise onto 293T cells, and incubated at 37°C. At 6 h after transfection, media were replaced with reduced-serum medium (Opti-MEM) containing 0.1% BSA. At 48 h after transfection, trypsin treated with N-tosyl-L-phenylalanine chloromethyl ketone (TPCK-trypsin; 0.5 μg mL^{-1}) was added. After incubation for 30 min at 37°C, supernatants were removed and cell debris was filtered with a 0.45-μm polyethersulfone (PES) syringe filter. The rescued IAVs were stored at –80°C until expansion in MDCK cells. For optimization of the reverse genetics conditions, we compared the

regular reverse genetics procedure conditions (gray) with modified conditions (**a-e**). Data are reported as mean ± SD (N= 3 for all experiments).

(**a**) Variation with pCAGGS plasmid; optimized condition, 0.5 μg pCAGGS plasmid.

(**b**) Variation with pHH21 plasmid; optimized condition, 0.6 μg pHH21 plasmid.

(**c**) Variation with transfection reagent (TransIT-LT1); optimized condition, 5 μL transfection reagent per μg total plasmid DNA.

(**d**) Variation with incubation condition; optimized condition, incubation with transfection reagent mixture alone for 20 min followed by incubation with transfection reagent mixture and added plasmid DNAs for 20 min.

(**e**) Variation with coculture condition of 293T and MDCK cells and time of adding TPCK-trypsin; optimized condition, coculture of 1×10^6 293T cells and 0.33×10^6 MDCK cells, replacing media with reduced-serum medium (Opti-MEM) containing 0.1% BSA at 6 h after transfection, adding TPCK-trypsin (final concentration, 0.5 μg mL^{-1}) at 24 h after transfection, and harvesting at 48 h after transfection.

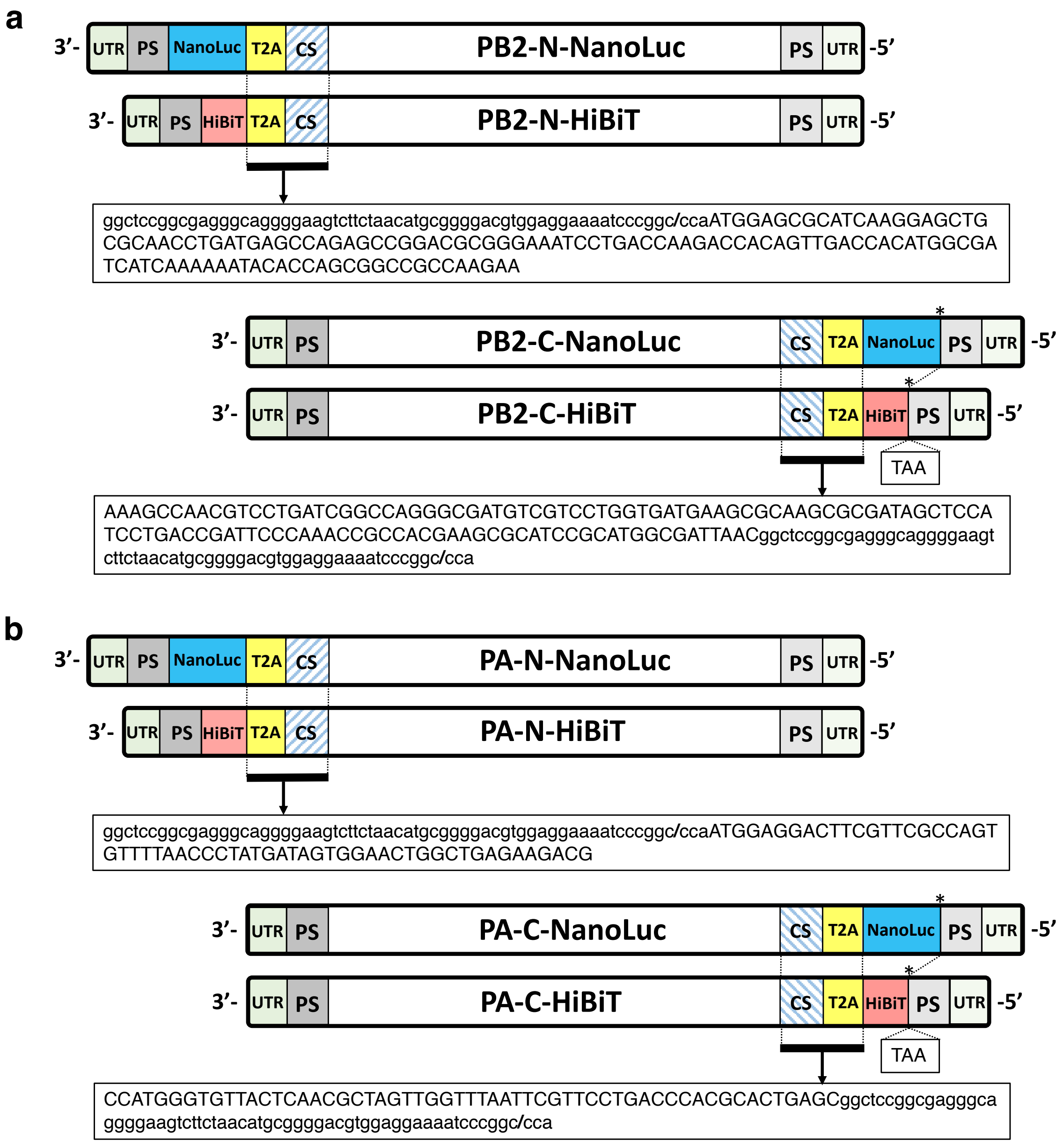

Supplementary Fig. 8. Modified sequences in PB2 (a) and PA (b) segments. Nucleotide sequences at 3'- or 5'- end of PB2 and PA segments are indicated: T2A sequence (lowercase letter), T2A cleavage site (/), codon swapped (CS) sequence (uppercase letter), and stop codon (*).

a.

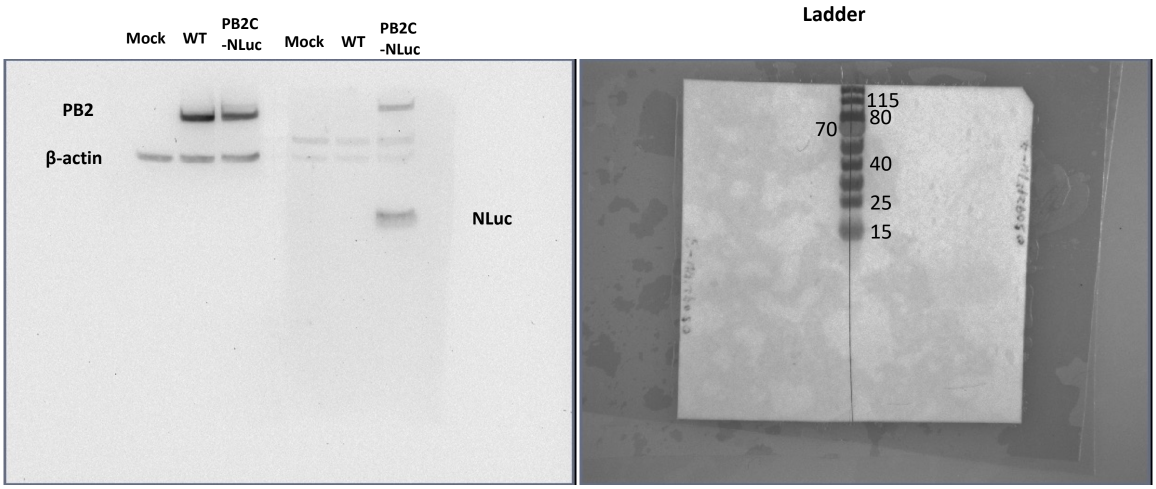
Ladder
Mock
WT
PB2C -NLuc
Mock
WT
PB2C -NLuc
PB2
β-actin
NLuc
115
80
70
40
25
15

b.

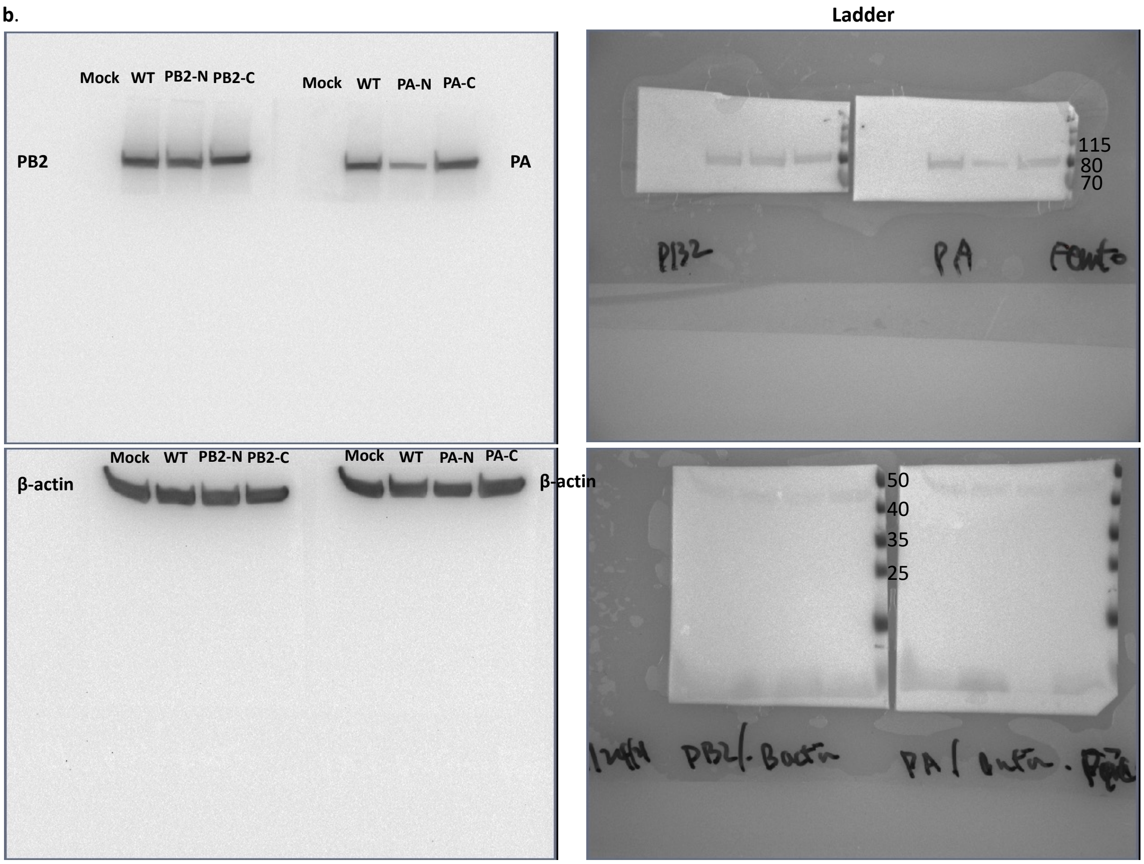
Ladder
Mock
WT
PB2-N
PB2-C
Mock
WT
PA-N
PA-C
PB2
PA
115
80
70
Mock
WT
PB2-N
PB2-C
Mock
WT
PA-N
PA-C
β-actin
β-actin
50
40
35
25

c.

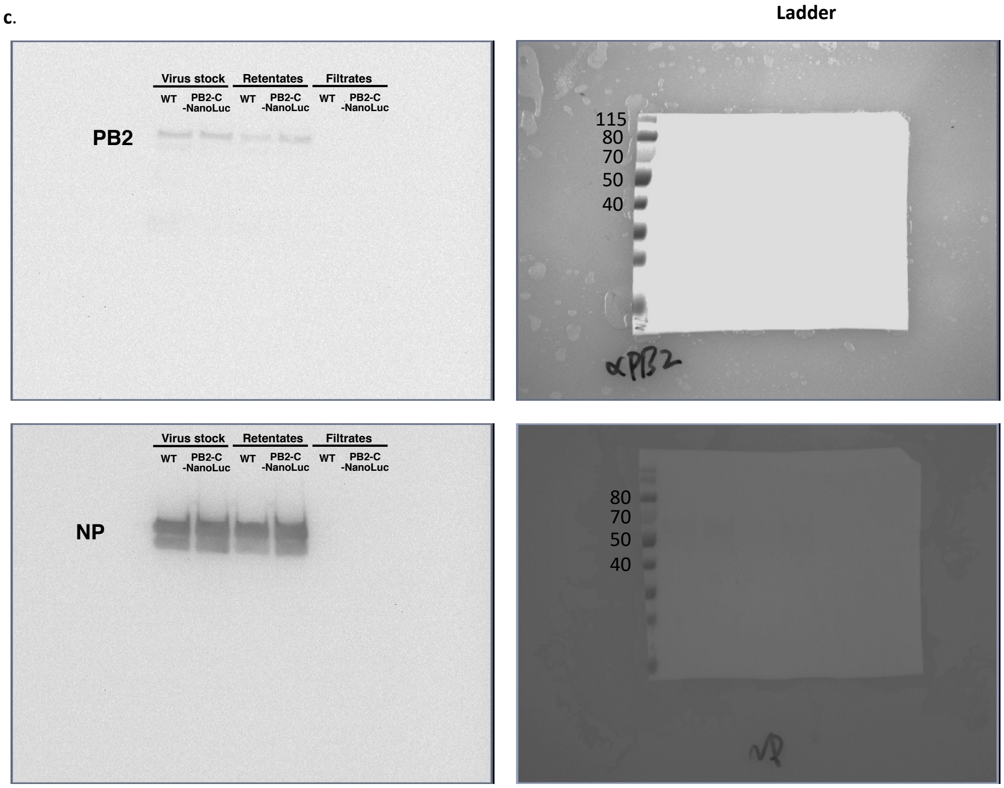
Ladder
Virus stock
Retentates
Filtrates
WT
PB2-C -NanoLuc
PB2
NP
115
80
70
50
40
αPB2

d.

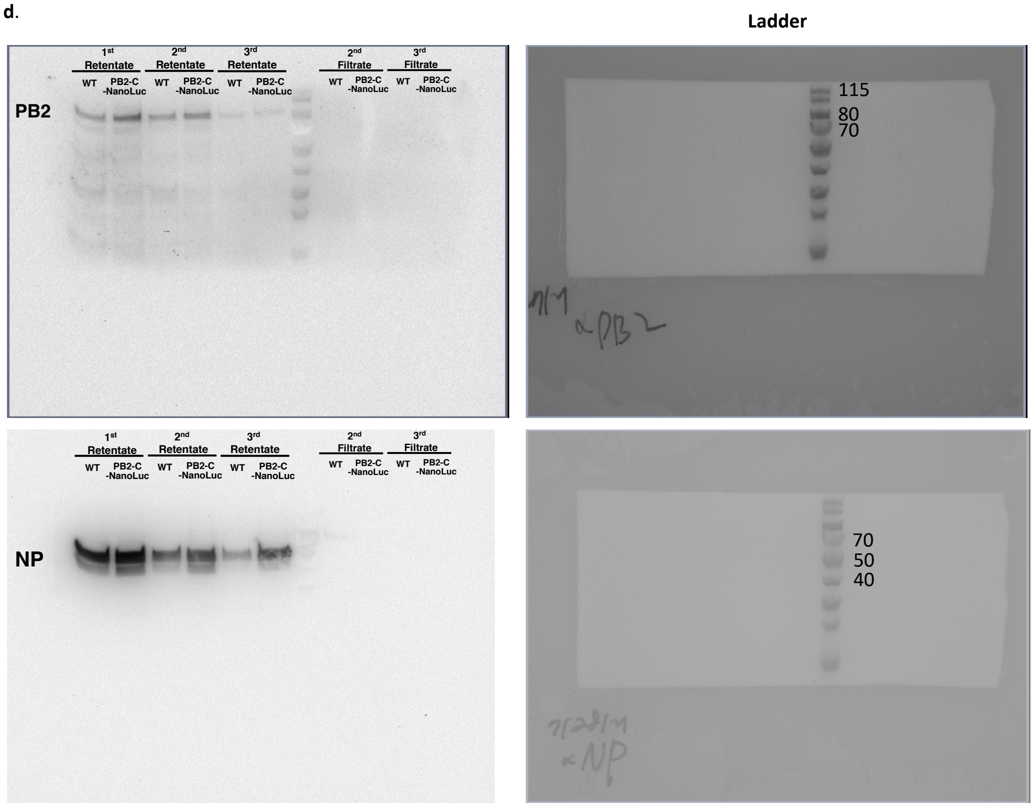
Ladder
1st Retentate
2nd Retentate
3rd Retentate
2nd Filtrate
3rd Filtrate
WT
PB2-C -NanoLuc
PB2
NP
115
80
70
50
40
αPB2
αNP

Supplementary Fig. 9. Original images of western blots before cropping.

(**a**) Full-length blots in Fig. 1d.

(**b**) Full-length blots in Fig. 1e, f.

(**c**) Full-length blots in Fig. 8d.

(**d**) Full-length blots in supplementary Fig. 5.

Supplementary Table 1. ***p*** **value of Pearson and Spearman correlation.**

		p *		*p* †
		Luciferase assay (Supernatant)	Luciferase assay (MDCK cells)	Flux
**2 PFU**	Plaque assay	0.0749	3.03E-13	0.0248
	Luciferase assay (Supernatant)		0.0508	0.5534
	Luciferase assay (MDCK cells)			0.0192
**20 PFU**	Plaque assay	0.0233	0.0410	0.0003
	Luciferase assay (Supernatant)		6.74E-07	0.0009
	Luciferase assay (MDCK cells)			0.0238
**200 PFU**	Plaque assay	0.0011	7.24E-07	0.0003
	Luciferase assay (Supernatant)		0.0042	0.0160
	Luciferase assay (MDCK cells)			0.1681
**2000 PFU**	Plaque assay	1.98E-07	7.11E-13	0.0004
	Luciferase assay (Supernatant)		1.78E-08	0.0001
	Luciferase assay (MDCK cells)			0.0189
**Nasal turbinate**	Plaque assay	0.2904	4.49E-09	0.0007
	Luciferase assay (Supernatant)		0.8496	0.0085
	Luciferase assay (MDCK cells)			0.0002
**Trachea**	Plaque assay	3.56E-09	1.70E-12	0.0058
	Luciferase assay (Supernatant)		4.30E-13	0.0047
	Luciferase assay (MDCK cells)			0.5742
**Lung**	Plaque assay	1.5651E-05	4.95449E-07	0.0403
	Luciferase assay (Supernatant)		4.12047E-06	0.0009
	Luciferase assay (MDCK cells)			0.0529

**p* value of Pearson correlation; †*p* value of Spearman correlation
